# DNA analysis of *Castanea sativa* (sweet chestnut) in Britain and Ireland: Elucidating European origins and genepool diversity

**DOI:** 10.1371/journal.pone.0222936

**Published:** 2019-09-25

**Authors:** Rob Jarman, Claudia Mattioni, Karen Russell, Frank M. Chambers, Debbie Bartlett, M. Angela Martin, Marcello Cherubini, Fiorella Villani, Julia Webb

**Affiliations:** 1 Centre for Environmental Change and Quaternary Research, School of Natural & Social Sciences, University of Gloucestershire, Cheltenham, United Kingdom; 2 Istituto di Ricerca sugli Ecosistemi Terrestri (IRET), Consiglio Nazionale delle Ricerche, Porano, Italy; 3 K Russell Consulting Ltd, Leighton Bromswold, Huntingdon, United Kingdom; 4 Faculty of Engineering & Science, University of Greenwich, Chatham Maritime, United Kingdom; 5 Department of Genetics, University of Cordoba, Cordoba, Spain; Aristotle University of Thessaloniki, GREECE

## Abstract

*Castanea sativa* is classified as non-indigenous in Britain and Ireland. It was long held that it was first introduced into Britain by the Romans, until a recent study found no corroborative evidence of its growing here before *c*. AD 650. This paper presents new data on the genetic diversity of *C*. *sativa* in Britain and Ireland and potential ancestral sources in continental Europe. Microsatellite markers and analytical methods tested in previous European studies were used to genotype over 600 *C*. *sativa* trees and coppice stools, sampled from ancient semi-natural woodlands, secondary woodlands and historic cultural sites across Britain and Ireland. A single overall genepool with a diverse admixture of genotypes was found, containing two sub groups differentiating Wales from Ireland, with discrete geographical and typological clusters. *C*. *sativa* genotypes in Britain and Ireland were found to relate predominantly to some sites in Portugal, Spain, France, Italy and Romania, but not to Greece, Turkey or eastern parts of Europe. *C*. *sativa* has come to Britain and Ireland from these western European areas, which had acted as refugia in the Last Glacial Maximum; we compare its introduction with the colonization/translocation of oak, ash, beech and hazel into Britain and Ireland. Clones of *C*. *sativa* were identified in Britain, defining for the first time the antiquity of some ancient trees and coppice stools, evincing both natural regeneration and anthropogenic propagation over many centuries and informing the chronology of the species’ arrival in Britain. This new evidence on the origins and antiquity of British and Irish *C*. *sativa* trees enhances their conservation and economic significance, important in the context of increasing threats from environmental change, pests and pathogens.

## Introduction

*Castanea sativa* (sweet chestnut) is presently classified as non-indigenous in Britain and Ireland, and until a recent re-evaluation [1] it was thought introduced to Britain during the Roman period (AD 43–410), and to Ireland sometime thereafter: a test of the ‘Roman introduction to Britain’ thesis has not found any corroborative evidence [1]. The role of natural and anthropogenic vectors for the expansion of various tree and shrub species from refugia since the Last Glacial Maximum (LGM) in Europe is increasingly being reviewed, including *C*. *sativa* [2], *Quercus robur* and *Q*. *petraea* [3] and *Corylus avellana* [4]. DNA analysis provides a powerful tool, additional to archaeological and palaeoenvironmental analyses, to track the movement of trees across the post-LGM landscape [5, 6].

### *Castanea sativa* in continental Europe

Palaeoenvironmental evidence indicated that *C*. *sativa* survived in refugia during the LGM in Iberia, S France, Italy, the Balkans, Greece, Turkey and the Caucasus [2, 7]. Genetic studies of ‘wild’ *C*. *sativa* populations in these areas [8, 9, 10, 11] have described these same refugia groups as three distinct genepools: ‘eastern’ (east Turkey to the Caucasus), ‘central’ (west Turkey, Greece, Bulgaria) and ‘western’ (Italy, S Switzerland, S France, Spain, Portugal). Recent studies [12, 13] have corroborated the indigenous status of *C*. *sativa* populations in parts of continental Europe and their derivation from these refugia; and described the redistribution of genotypes via natural colonization and anthropogenic translocation. *C*. *sativa* was used for wood and fruit production from before the Classical Greek and Roman periods, when it was translocated across southern Europe; and was then more intensively cultivated by monastic, royal and noble estates, by farmers and by foresters across Europe [14, 15, 16, 17, 18].

### *Castanea sativa* in Britain

**Fig 1:**
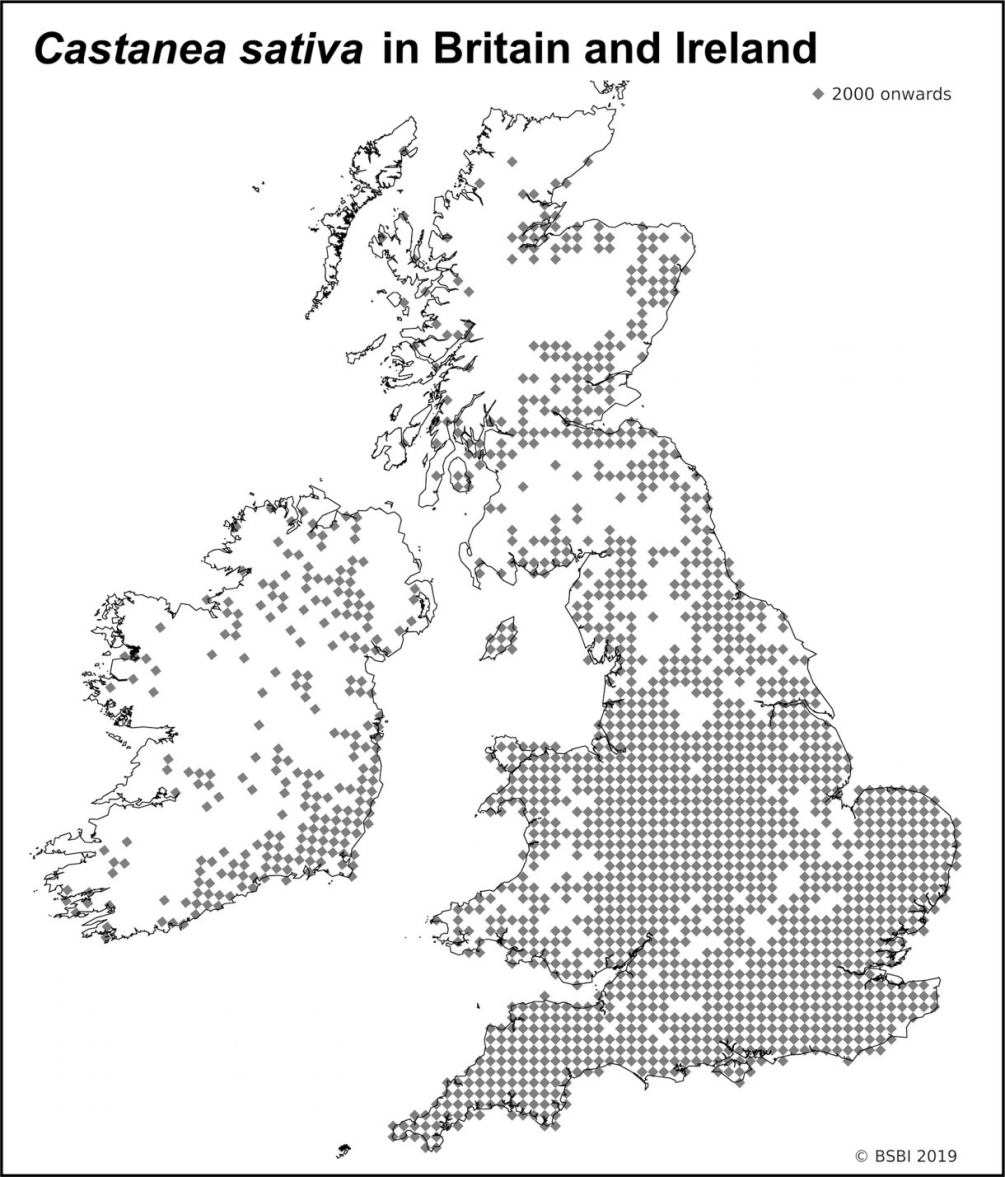
Distribution of *Castanea sativa* in Britain and Ireland.

*C*. *sativa* has been classified in accounts of the British flora as an archaeophyte of Roman introduction [19, 20, 21, 22]: ‘an honorary native’ [23] is how it is typically described in some ancient semi-natural woodland contexts. *C*. *sativa* trees are presently recorded growing across most of Britain (Fig 1) [24], but *C*. *sativa* woodland is only locally abundant where slightly acidic (pH 4.5–5.5) soils over moist but well-drained substrates predominate [25].

There are approximately 28,200 ha of *C*. *sativa*-dominated woodland in Britain [26], located predominantly in the east, south and west regions of England, occurring as coppice and high forest in ancient semi-natural woodlands and secondary woodlands. *C*. *sativa* can presently set viable seed and self-regenerate as far north as Sutor, Cromarty, Scotland (57°40’N, 4°01’W) (Diane Gilbert 2018 pers. com.).

In many historic landscapes in Britain, sweet chestnut trees and pollards are amongst the oldest and largest of any species, situated in wood pastures, medieval deer parks, historic designed parks and gardens, and on ancient boundaries within farmed landscapes [23]. In Britain there are over 1,400 individual ancient *C*. *sativa* trees of >6 metres girth (measured at 1.3–1.5 m above the root collar) [27].

Despite its oft-asserted status as a Roman archaeophyte, only nine acceptable records of *C*. *sativa* remnants have been found in Britain up to AD 650 (including only one find of nut fragments): none could be verified as grown in Britain [1]. The early history of *C*. *sativa* growing in Britain is unknown, despite three centuries of speculation [23, 28]. The earliest written record of *C*. *sativa* growing in Britain presently dates from AD 1113 [29]: this and two other written records from the 12^th^ and 13^th^ centuries AD indicate specific *C*. *sativa* trees and woods that were established in at least three places in southern England and SE Wales sometime before the 12^th^ century AD [1]. The oldest extant British *C*. *sativa* trees that have been dendrochronologically cross-dated presently date from *c*. AD 1640 [30].

### *Castanea sativa* in Ireland

*C*. *sativa* is conventionally presumed to have been introduced to Ireland sometime in the medieval period [31, 32]. The largest extant *C*. *sativa* tree in Ireland is the 10.78 m girth ‘Wesley tree’ (Rossanna, Co. Wicklow); thirty-eight ancient *C*. *sativa* trees are recorded for Eire [33]. In Northern Ireland, there are twenty-two ancient *C*. *sativa* trees >6 m girth [27]. In Eire, Coillte (the State-sponsored forestry company) manages *c*. 100 ha of *C*. *sativa* woodland (*c*. 95% of the *C*. *sativa* woodland in Eire), mostly in the southern and south-eastern counties (Ted Horgan 2017 pers. com.)–Fig 1.

### Genetic analysis of *Castanea sativa* in Britain and Ireland

Despite many studies of *C*. *sativa* across Europe [12, 13], British and Irish populations have largely been ignored. Two previous studies assessed a few samples from *C*. *sativa* trees in England: six coppice groups in southern England [34] were used in the European CASCADE project to establish reference SSR markers for *C*. *sativa* [35, 36, 37] and subsequently [38]; and some preliminary data from the present research were included in an Europe-wide landscape genetics study of *C*. *sativa* [11].

### Aims of the paper

This paper aims to describe the genetic diversity of *C*. *sativa* trees in Britain and Ireland, to discover any geographical and typological associations and, by comparison with *C*. *sativa* genotypes from sites in continental Europe, indicate potential ancestral sources. Genetic information can locate close kinships and clonal groups of plants; and help to determine the antiquity and management history of individual trees and coppice stools. The combined information on genetic diversity, relatedness and antiquity can help elucidate whence *C*. *sativa* arrived in the British Isles, and possibly when.

## Materials and methods

*Castanea sativa* trees were sampled from sites across Britain and Ireland, which were selected to represent a broad range of ecological, cultural and geographical contexts. Plant material for DNA analysis was collected from ancient trees and coppice stools in ancient semi-natural woodlands and historic cultural sites in England and Wales, hereinafter referred to as the ‘Historic trees’ sample set; and from trees and coppice of mixed antiquities selected from sites included in the Future Trees Trust (FTT) Sweet Chestnut Improvement Programme [39], which covers England, Wales, Scotland and Ireland, hereinafter referred to as the ‘FTT trees’ sample set.

### Sampling strategy and site selection

For the ‘Historic trees’ samples, fresh leaf samples were collected from *Castanea sativa* plants in ancient semi-natural woodlands and historic non-wooded landscapes (‘plant’ here means ‘standard’ single-trunked trees, multi-stemmed trees including pollards and multi-stemmed ‘coppiced’ trees growing from ‘stools’) [40]. These ‘historic’ sites were identified from a range of sources, including archives and published contemporary accounts; the Ancient Tree Inventory [27]; maps of ancient semi-natural woodlands; designated historic monuments, parks and gardens; Forestry Commission and National Trust records; and peer review. A preliminary survey in 2013 tested the methods, followed by extensive sampling across England and Wales during 2014–2018. Wherever possible, several trees were sampled within each site to represent its characteristics: within an ancient woodland coppice, several discrete stools were sampled from different parts of the wood, and some stools were multi-sampled (to test for clonality); within an historic parkland, several ancient trees were sampled from specific features such as an avenue or grove, and some individual trees were multi-sampled (to test for grafting or bundle planting). For some historic parkland/garden sites, where only a single ancient *C*. *sativa* tree existed, samples were taken from several parts of the tree to test its genetic integrity. Several historic trees and coppice stools were re-sampled in consecutive years to test whether the analysis of leaves from the same stem but in different annual growth seasons produced consistently replicable results. Several trees were re-sampled by collecting dormant buds (during winter) instead of leaves, to check their replicability.

For the ‘FTT trees’, fresh leaf samples were collected from the FTT clonal archive of plants, which had been established from outstanding individual timber trees and coppice stools located in long-established woodland (not recent plantations) [39].

### DNA extraction and Microsatellite analysis

For direct comparison and integration of British and Irish samples with samples from an earlier Europe-wide study [11], the analytical procedures and materials used were identical with that study. Eight polymorphic microsatellite markers developed and used for *Castanea sativa* [10, 34, 35] (CsCAT-1, -2, -3, -6, -14, -16; and EMCs-25, -38) were used to genotype the British and Irish samples: six of these markers had been used by the European study [11] (CsCAT-1, -3, -6, -16; and EMCs-25, -38). Multiplex-PCRs were performed exactly as described in that study: the samples were run on an ABI Prism 3130 Avant DNA sequencer and the resulting raw data were analyzed using GeneMapper software (Life Technologies). The alleles were determined by automated binning and checked by visual inspection.

### Data analysis

The microsatellite data were used to estimate genetic diversity, to determine whether the British and Irish samples constituted a single genepool or several genepools: the British and Irish samples were then integrated with the European dataset [11] and analysed to investigate the potential continental European origin(s) of the British and Irish germplasm. The first step was to identify discrete genotypes from all the samples, using GenAlEx 6.51b2 Multilocus Matching Programme, so that the diversity analyses could be run without any matching samples. Groups of matching samples were assessed separately for clonal characteristics.

### Genetic diversity and population structure

Null allele frequencies were estimated for each locus and population using FreeNA [41]. Allelic richness (Ar) and private alleles (PAr) were calculated by the statistical method of rarefaction to avoid bias due to different sampling size, implemented by the software HP-Rare 1.1 [42]. Genetic diversity parameters were measured using the software GenAlEx 6.51b2 [43]: observed (Na) and effective (Ne) number of alleles, observed (Ho) and expected (He) heterozygosis [44], unbiased estimate of mean expected heterozygosis (UHe) and the diversity index of Shannon (I) were computed; and the inbreeding coefficient (F_IS_) was derived using GENEPOP 4.2 software Option 5 [45]. The values of the population differentiation coefficient (F_ST_) were assessed using FreeNA [41] to detect genotypic differences, employing ENA correction for null allele bias and F_ST_ overestimation. A threshold for determining relevance of F_ST_ estimates was derived from upper and lower (10% and 90%) percentiles of the dataset of pairwise values, to indicate ‘high’ and ‘low’ differentiation relative to the actual range of genetic difference in the populations observed. AMOVA was performed using GenAlEx 6.51b2.

The genetic distance (GD) among individuals and the Nei genetic distance among groups of individuals were calculated using GenAlEx 6.51b2; Principal Coordinates Analyses (PCoA) were run to test various parameters that might define postulated populations. Unweighted Neighbour Joining Cluster analysis (UPGMA) used DARwin ver.6 [46] to construct a dendrogram of hierarchical relationships between samples.

Pairwise relatedness (*r*) was estimated using Queller and Goodnight [47] and Lynch and Ritland [48] analyses run in GenAlex v.6.51b2, to detect kinships within and between populations. The Lynch and Ritland method (LRM) states that an *r* value close to the maximum calculable 0.5 indicates an identical twin, 0.25 indicates a full sibling relationship (parent–offspring, or siblings sharing the same parents), and 0.125 indicates a half sibling (one shared parent). In the Queller and Goodnight method (QGM), an *r* value close to the maximum 1, 0.5 and 0.25 correspond to identical twin, full sibling/parent-offspring, and half sibling, respectively.

The samples were analysed using GenAlEx 6.51b2Multilocus Matching programme to identify samples with matching multilocus genotypes for codominant data, at two hierarchical stages of division: samples matching exactly at all loci (‘clonal’), and matching at all loci except one (a ‘near match’). The analysis was run for the 8–SSR dataset and then separately for the 6–SSR dataset. Unexpected results (based on the tree/stool descriptions) were checked by visual assessment of the GeneMapper automated binning output, followed by repeat analyses of leaf material from that sample. Samples recorded as ‘near matches’ were tested for the degree of allelic difference; and checked on site for evidence of direct biological linkage between potentially identical clonal plants (in biological terms, between a ‘genet’ and a putative ‘ramet’). Where only a single allele difference at a single locus was recorded (such as ‘232’ instead of ‘230’), a somatic mutation from the parent plant (genet) was accepted and the sample pair was considered ‘clonal’. All ‘near matches’ greater than a single allele difference (‘234’ instead of ‘230’) were rejected as ‘non-clonal’. These results were compared with the *r* pairwise relatedness results (LRM and QGM) to check the compatibility of these methods for determining very close or clonal relationships.

A Bayesian analysis (STRUCTURE v.2.3.4 [49]) was run with the option of including prior information on the spatial location of populations, using the admixture model, with the number of tested clusters (*K*) from 1 to the purported number of provenances plus 2. Six independent runs were performed for each *K* value, with a burn-in period of 10^3^ steps followed by 10^5^ MCMC replicates. The analysis assigns individuals into each of the *K* clusters based on the membership coefficient (*Q*-value). To identify the number of clusters that best explained the data, the rate of change on L(*K*) (termed *ΔK*) between successive *K* values was calculated [50] using STRUCTURE HARVESTER software [51]. The six runs for each simulation were averaged using CLUMPP and displayed using DISTRUCT, provided by CLUMPAK [52]. To delineate genetic repartition of sweet chestnut populations, a phylogenetic dendrogram UPGMA [53] was constructed, using Nei genetic distance corrected for F_ST_ using the software POPTREE2 [54] and drawn with FigTree v1.4.3 [55].

### Site characterisation for British and Irish samples

Five parameters were selected to provide geographical, ecological and cultural frameworks within which to analyse the British and Irish genotypes for any spatial or typological pattern of distribution: Ordnance Survey 100km square Map Grid; Administrative Counties and Regions; Seed Zones [56]; Cultural landscapes [57]; and Site Types (see below). Each parameter dataset was analysed using GenAlEx 6.51b2 to generate a ‘genetic distance by population’ table, PCoA chart and UPGMA dendrogram. GENEPOP 4.2 produced F_IS_ values of the inbreeding coefficient for each site, and F_ST_ estimates of pairwise differentiation between sites.

## Results

In total, 753 samples were collected from 259 sites across Britain and Ireland (S1 File; S1 Table); in addition, 22 samples were collected from the Tortworth Chestnut tree (South Gloucestershire, England) for clonal analysis and antiquity tests. Five types of site were identified in the survey: ‘Type A’ (high forest standard trees < *c*. 200 years old); ‘Type B’ (coppice stools < *c*. 200 years old); ‘Type C’ (ancient coppice stools in ancient semi-natural woodland > *c*. 200 years old); ‘Type D’ (ancient trees in woods and historic landscapes > *c*. 200 years old); and ‘Type E’ (historic garden trees, typically singular and of great antiquity). These Site Types are fully described in S3 File (para 5).

### Genetic diversity of British and Irish *Castanea sativa* trees

The eight microsatellite loci comprised 91 alleles: CsCAT-2, -3, -6 and EMCs38 had the highest number of alleles (A and Ae) and values of genetic diversity (He); EMCs25 showed the lowest number of alleles (5), low values of Ho and high values of F_IS._ FreeNA check for null alleles showed EMCs25 consistently produced null alleles.

Of the 753 samples, 611 were identified as discrete genotypes (GenAlEx 6.51b2 Multilocus Matching Programme) and used for the diversity analyses, for Britain and Ireland as a single site group and for three separate site groups ‘England’, ‘Ireland’ and ‘Wales’ (Table 1); 88 groups of matching samples were identified for separate clonal analyses.

**Table 1:** Genetic diversity measures for *Castanea sativa* in Britain and Ireland.

Population	N	A	Ae	I	Ho	He	UHe	Ar	PAr	F_IS_ P<0.001	F_ST_ without ENA	F_ST_ with ENA
**England**	516	14.5	4.957	1.792	0.713	0.74	0.741	10.07	0.97	0.041	0.0081	0.0083
**Ireland**	48	10	4.431	1.669	0.661	0.7	0.708	9.97	0.89	0.062	0.006	0.0057
**Wales**	47	9.25	4.102	1.604	0.657	0.706	0.713	9.25	0.56	0.074	0.0141	0.0138
**Britain & Ireland**	611	14.75	4.915	1.69	0.704	0.737	0.738	14.75	–	0.05		

N = Number of samples, A = Mean Number of different alleles, Ae = Mean Number of effective alleles, I = Shannon’s Information Index, Ho = Observed heterozygosity, He = expected heterozygosity, UHe = Unbiased expected heterozygosity, Ar = allelic richness (average over loci), PAr = private allelic richness (average over loci), F_IS_ = inbreeding coefficient, F_ST_ = genetic differentiation, ENA = FreeNA estimate of null allele frequency per locus.

Comparable values of genetic diversity parameters were observed for England, Ireland and Wales; slightly higher values of allelic richness (Ar) and private allelic richness (PAr) were observed for England samples. F_IS_ was higher for Wales (0.074) than for England or Ireland, as also was F_ST_ (Wales 0.014), indicating a relatively higher degree of inbreeding and isolation for Wales sites. AMOVA analysis of the England, Ireland and Wales groups of samples (S2 Table) found the majority of variability (95%) within individuals, with 4% amongst individuals and 1% amongst groups.

### Genetic structure

**Fig 2:**
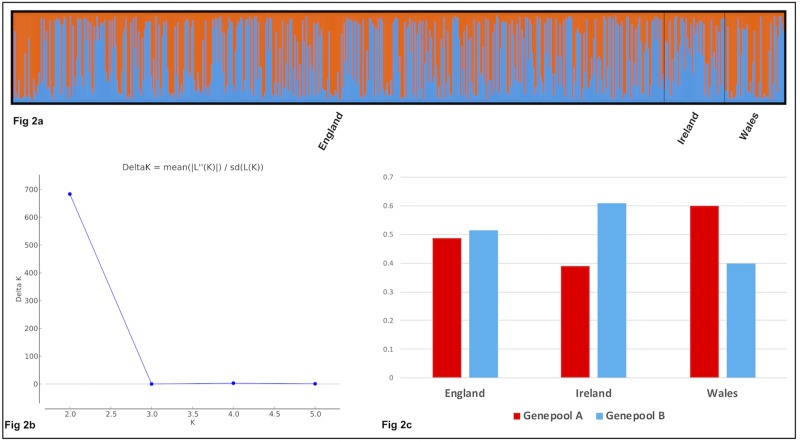
STRUCTURE analysis of England, Ireland and Wales (611 samples, 8 SSRs): (2a) ‘Genepool A’ (red) and ‘Genepool B’ (blue); (2b) K = 2 (Evanno); (2c) differentiation between Ireland and Wales in ‘Genepool A’ and ‘Genepool B’.

The genetic structure of England, Ireland and Wales samples was revealed by three statistical approaches:

1) The Bayesian analysis implemented by STRUCTURE indicated the presence of two genepools, ‘Genepool A’ and ‘Genepool B’ (K = 2 Evanno, Δ*K* = 700)–Fig 2.

**Fig 3:**
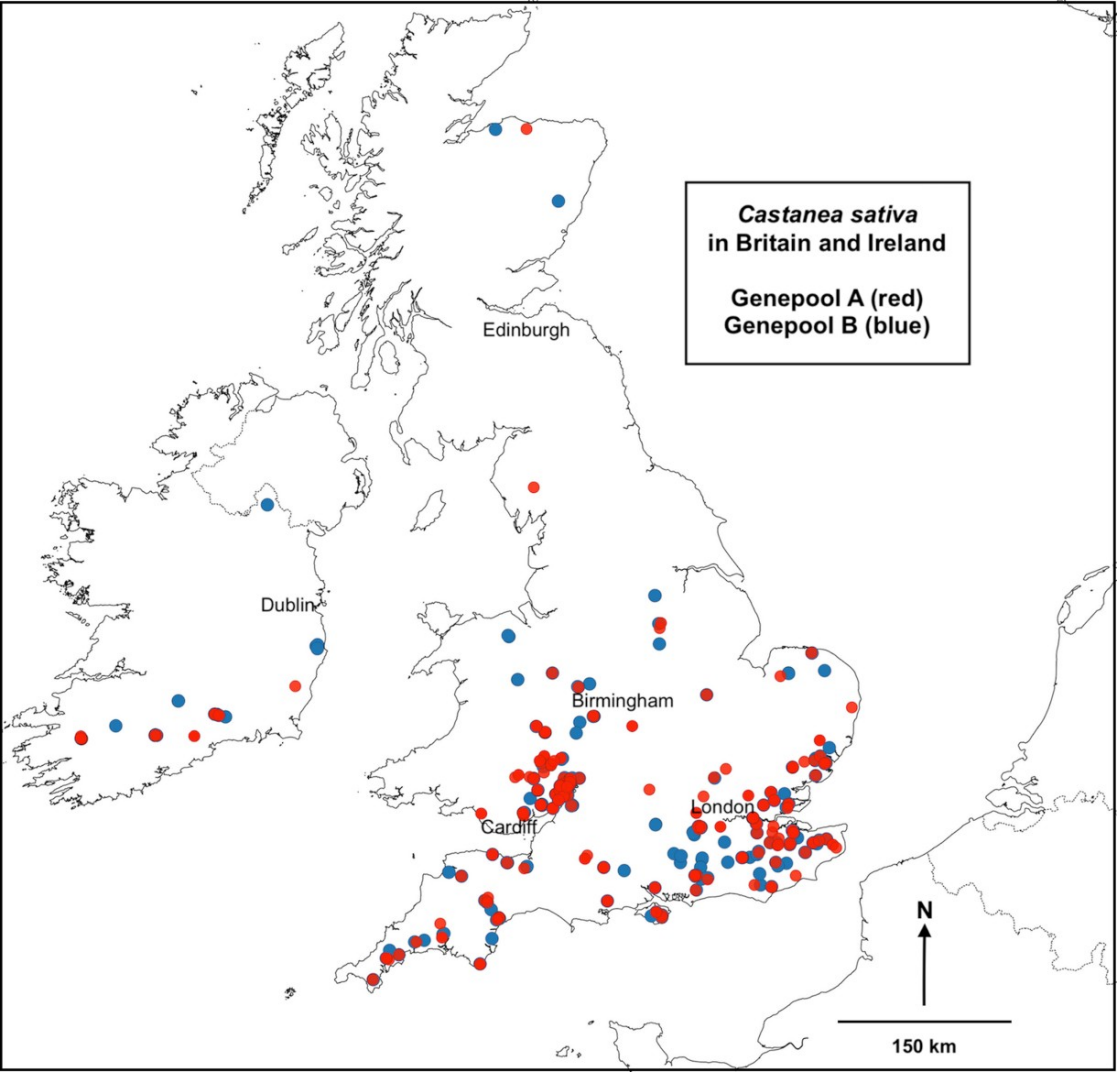
**England, Ireland and Wales samples in ‘Genepool A’ (red) and ‘Genepool B’ (blue).** Map base open-sourced from ‘EuroGeographics and UN-FAO @EuroGeographics’.

The England, Ireland and Wales samples were mapped according to their ‘Genepool A’ or ‘Genepool B’ membership–Fig 3 and S2 File (for viewing in GIS mapping). Local and regional spatial clusters of ‘Genepool A’ and ‘Genepool B’ sites were evident, indicating local area fidelity, but overall there was no evidence of broad geographical separation between the two genepools.

**Fig 4:**
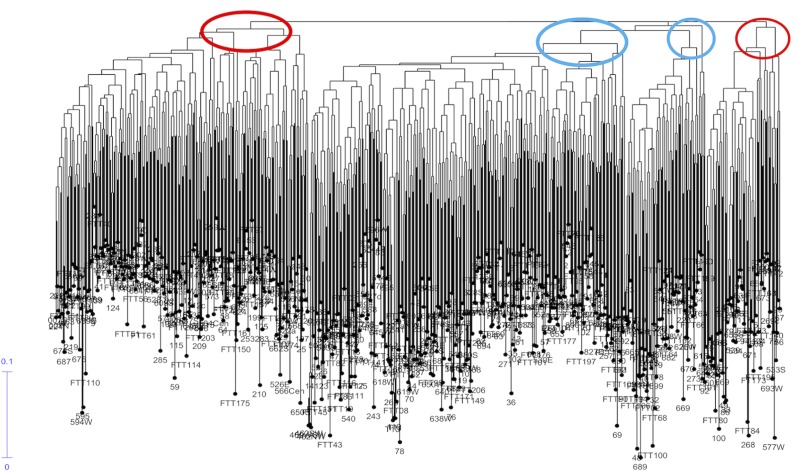
Dendrogram for England, Ireland and Wales samples (Nei genetic distance) indicating two major and two minor clades (‘Genepool A’–red; ‘Genepool B’–blue).

2) The dendrogram of Nei genetic distance, using the Unweighted Neighbour Joining Method (DARwin ver. 6), indicated two main clades and two minor clades: genetic distance among individuals was low–Fig 4 (see S1 Fig for expandable image).

Some samples from the same site clustered together, whilst other samples from the same site dispersed through the dendrogram: ‘top down’ linkages within and between some sites were identifiable. Comparison of the dendrogram with the STRUCTURE output allowed matching of ‘Genepool A’ and ‘Genepool B’ members to the two main clades and to the two minor clades of the dendrogram, shown ringed in red (Genepool A) and blue (Genepool B) on Fig 4, such that the dendrogram depicts three main clades, with ‘Genepool A’ to the left, ‘Genepool B’ centrally in 2 clades, and ‘Genepool A’ minor clade to the right.

**Fig 5:**
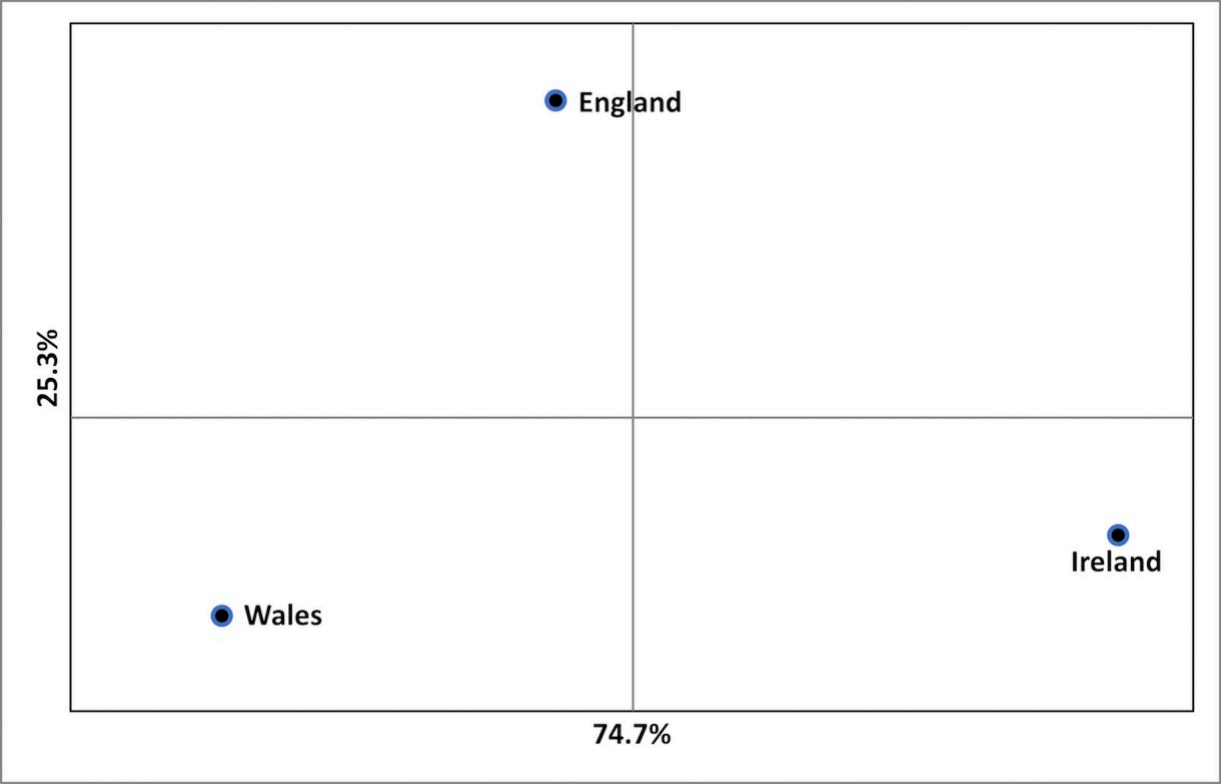
PCoA plot of samples from England, Ireland and Wales, showing differentiation between Wales and Ireland.

3) PCoA evaluation of the England, Ireland and Wales groups of samples corroborated the differentiation between Wales and Ireland, with a high degree of separation on the *x*-axis (74.7%)–Fig 5.

### Relatedness of the England, Ireland and Wales samples and sites

Three measures were used to test for relatedness between samples and between sites: *F* statistics, relatedness estimation (*r*) and clonal analysis. Pairwise F_ST_ estimates, using a threshold value of F_ST_ = 0.05, found no great differentiation (all scores were <0.05) between the three country groups, with the highest differentiation between Wales and Ireland (F_ST_ = 0.016). Pairwise F_ST_ estimates using the five parameter groups (S3 File; S3 Table) found no differentiation between any of the samples at F_ST_ >0.05 for any of the five parameters, except in the ‘Counties’ dataset, where 22 out of 300 (7.3%) sample pairs measured >0.05, to a maximum 0.093, again differentiating between Wales and Ireland sites. Some sites (including iconic historic trees at Bushy House, Seven Sisters Penshurst and the Tortworth Chestnut) had values of F_ST_ >0.15 across all five parameters, and were highly differentiated from all other British and Irish sites. F_IS_ values were estimated for each site with >2 samples (S3 Table): they ranged from –0.857 (Warren Plantation A, Essex) to 0.467 (America Wood, Isle of Wight).

Kinship *r* estimation between individual samples, using pre-selected values of *r* >0.4 (LRM) and >0.75 (QGM) to define a threshold above which samples were deemed to have very close kinship, identified the ‘highest related’ pairs of samples (S4 Table); these were corroborated by the pairwise F_ST_ and clonal ‘near matches’ analyses. For example, 73 samples from coppice stools and ancient trees in ‘Castiard’ (Forest of Dean, Gloucestershire) that had been analysed as clonal groups produced pairwise *r* scores of 0.500 for LRM and 1.000 for QGM; and very close kinship scores were obtained for ‘near match’ samples (up to 0.383 in LRM and 0.924 in QGM)–S5 Table. Some of the very close kinship samples from across Britain grew on adjacent trees/stools, but some were from widely disjunct trees (such as between Croft Castle in Herefordshire and Seven Sisters Penshurst in Kent; and between Godinton in Kent and Hagley Hall in Worcestershire), perhaps evincing historical vegetative and/or seed translocation at an inter-regional scale.

Clonal analysis determined the relatedness of samples between and within sites: from across England and Wales, 185 samples formed 88 clonal groups. Thirty samples formed 15 pairs of ‘near matches’, four of which differed by a single allele at a single locus, so could be presumed somatic mutations and re-classified as clones. This clonal analysis of tree and stool components enabled the definition of the ‘genotypic’ size of large-girthed coppice stools and multiple-stemmed/collapsed trees, as opposed to their ‘visual’ size, thereby indicating their true antiquity. For example, a 16 m girth ring-form stool in Welshbury Wood was found genetically identical in all its parts, so is formed of a single-genotype original plant that has grown over centuries of repeated cutting into a very large stool; whereas a 15 m girth ring-stool growing nearby, which visually/structurally appeared like a single entity, was found to consist of three genetically different plants, indicating that it was probably much younger than the ‘genetically entire’ stool. A massive ancient tree at Baldwyns (Kent) appeared visually to be a single tree base with four main trunks with a basal girth of 12 m, but clonal analysis showed it was at least four genetically separate plants (one of which was a ‘near match’ with a large coppice stool in ancient woodland 10 km distant). In another case, two trees growing hundreds of metres apart (at Abenhall, Gloucestershire) were found to be clonal and so were presumed translocations of vegetative material. Two examples of grafted trees were revealed on two sites in England. The clonal information was used to confirm the antiquity of ancient trees and stools in the Site Type classification, below.

### Site-based relationships within the British and Irish genepool

The England, Ireland and Wales samples were sorted into five parameter-defined groups. In the four geographically defined groups, the PCoA and UPGMA analyses indicated genetic differentiation between samples in Ireland and Wales. In the fifth group, the Site Type evidence of tree/stool size and site history indicated that older (>200 years) trees and stools (Types C and D) were genetically differentiated from younger (<200 years) trees and stools (Types A and B). Type B (younger coppice) was the most differentiated of the five Site Types: STRUCTURE analysis placed it predominantly (64%) within ‘Genepool B’. Ancient historic garden trees (Type E) were differentiated by the PCoA from other ancient trees (Type D) as well as from the younger sites (Types A and B): STRUCTURE analysis placed Type E trees predominantly (60%) within ‘Genepool A’. The detailed results from the five parameter analyses are presented in S3 File.

The ‘Historical trees’ group was compared with the ‘FTT trees’ group: no genetic differentiation was found.

### European dimension for the British and Irish samples

The six SSR markers used for the previous European research [11] were applied to the British and Irish samples, identifying 608 discrete genotypes (560 from Britain, 48 from Ireland) for integration and comparative analysis with the continental European samples. S6 Table contains the full dataset (1332 samples, 41 site groups) of continental European, British and Irish samples.

### Genetic diversity analysis of the European samples

The continental European, British (subdivided into England and Wales) and Irish dataset, comprising 1332 samples in 41 site groups, was assessed for genetic diversity (S7 Table). The number of alleles (Na) and number of effective alleles (Ne) were greater for the England, Ireland and Wales groups than for any of the continental European groups; as were the values of I and He and allelic richness (Ar). Private allelic richness (PAr) was higher in Wales than in 61% of the continental European groups and in Ireland higher than in 44% of those groups. Null allele frequency estimation showed EMCs25 produced null alleles. Molecular variance was 11% among populations, 6% within groups and 83% within populations (P< 0.001). The combined continental European, British and Irish dataset produced a F_IS_ value of 0.067 and F_ST_ value of 0.114 (F_ST_ with ENA was 0.108).

### STRUCTURE analysis of the continental European, England, Ireland and Wales samples

For an initial STRUCTURE analysis, the England, Ireland and Wales samples were incorporated into the original full dataset of continental European populations (eastern and western) [11], comprising 2199 samples from 319 site groups. The results previously published [11] were confirmed, indicating a clear separation of ‘eastern’ from ‘western’ European site groups, with no connection between British and Irish sites and the ‘eastern’ European sites (S4 File).

**Fig 6:**
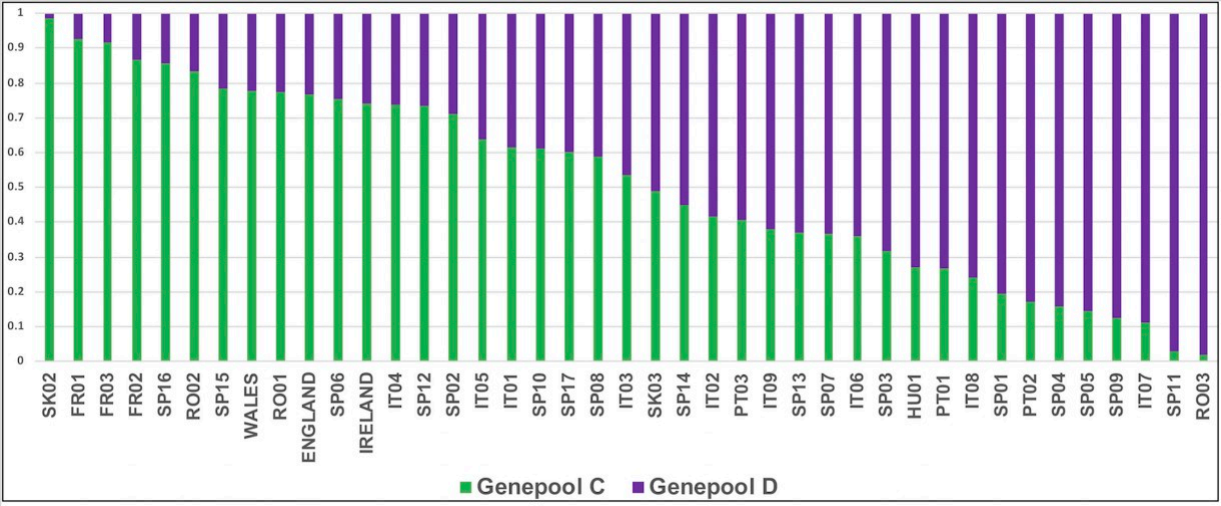
Continental Europe, England, Ireland and Wales sites, STRUCTURE output defining ‘Genepool C’ (green) and ‘Genepool D’ (purple).

**Fig 7:**
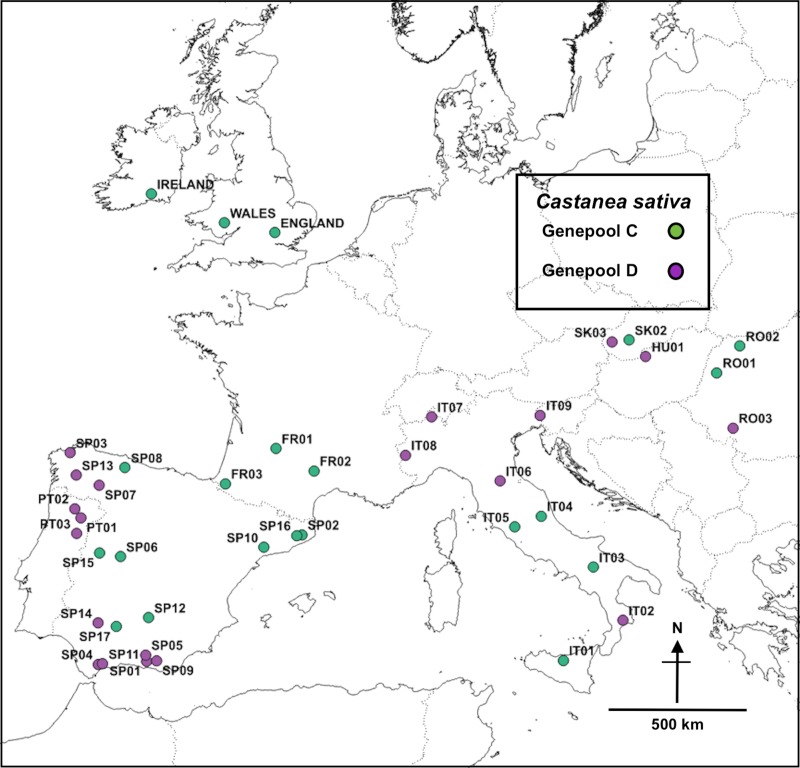
**Map of European sites, STRUCTURE output ‘Genepool C’ (green) and ‘Genepool D’ (purple).** Map base open-sourced from ‘EuroGeographics and UN-FAO @EuroGeographics’.

A ‘western’ European sites subset was defined, containing the British and Irish samples with Portugal (PT), Spain (SP), France (FR), Italy (IT) and some sites from Slovakia (SK), Romania (RO) and Hungary (HU): it comprised 1332 samples in 41 groups (38 continental European groups plus ‘England’, ‘Ireland’ and ‘Wales’). STRUCTURE analysis identified two main groups (K = 2 Evanno), termed ‘Genepool C’ and ‘Genepool D’: each site was allocated to ‘Genepool C’ or ‘Genepool D’ by the ‘K2-pop’ CLUMPP output, as shown in Fig 6, using Q = 0.5 as a threshold; and mapped (Fig 7; S5 File for GIS data in .kml format).

**Fig 8:**
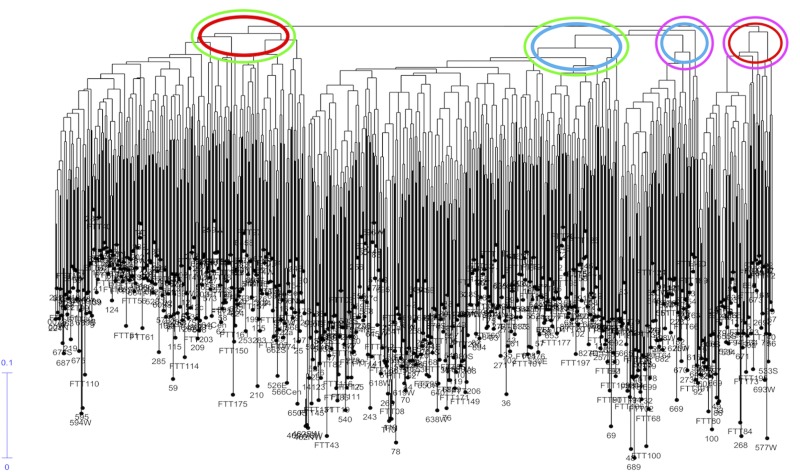
Dendrogram for England, Ireland and Wales samples (Genepool A–red; Genepool B–blue) overlain with their continental European classification into ‘Genepool C’ (green) and ‘Genepool D’ (purple).

The England, Ireland and Wales samples fitted predominantly (78%) within ‘Genepool C’, whereas in the Britain and Ireland analysis (using 8 SSRs) they had divided almost equally into two groups (‘Genepool A’ and ‘Genepool B’)–Fig 8.

STRUCTURE analysis of the ‘western’ European groups indicated that ‘Genepool C’ sites in England, Ireland and Wales derived predominantly from northern Iberia, southern France, central/southern Italy and northern Romania; whereas ‘Genepool D’ sites in England, Ireland and Wales derived predominantly from western and southern Iberia, northern Italy and Hungary, Romania and Slovakia. ‘Genepool D’ affiliations were stronger for Wales (notably with PT03, SP07, SP14) than for Ireland, so genetic differentiation between Wales and Ireland was again evinced. Typologically, Site Type E (ancient historic garden trees) was predominantly affiliated with ‘Genepool D’ (PT01, PT03, SP07), whereas the other four Site Types (A–D) were more affiliated with ‘Genepool C’. (Table 2).

**Table 2:** Summary of dominant relationships between continental European and England, Ireland and Wales sites, as defined by Pairwise F_ST_ scores <0.05.

Continental European sites	Genepool	Dominant relationship -England/Ireland/Wales	Dominant relationship—site type
PT01	D	England	E–ancient historic garden trees
PT03	D	Wales/England	C, D, E—Ancient trees and stools including historic garden trees
SP06	C	Wales	No differentiation
SP07	D	Wales/England	D, E—ancient trees and historic garden trees
SP12	C	Ireland/England	C, D—ancient trees and coppice
SP14	D	Wales	No differentiation
FR01	C	Ireland/England	A, D—trees (ancient and modern), but not coppice
FR02	C	Ireland/England	A, B—modern trees and coppice
FR03	C	Wales/England	all types
IT05	C	England	B—modern coppice
RO01	C	Wales/England	all types

### The UPGMA analysis

**Fig 9:**
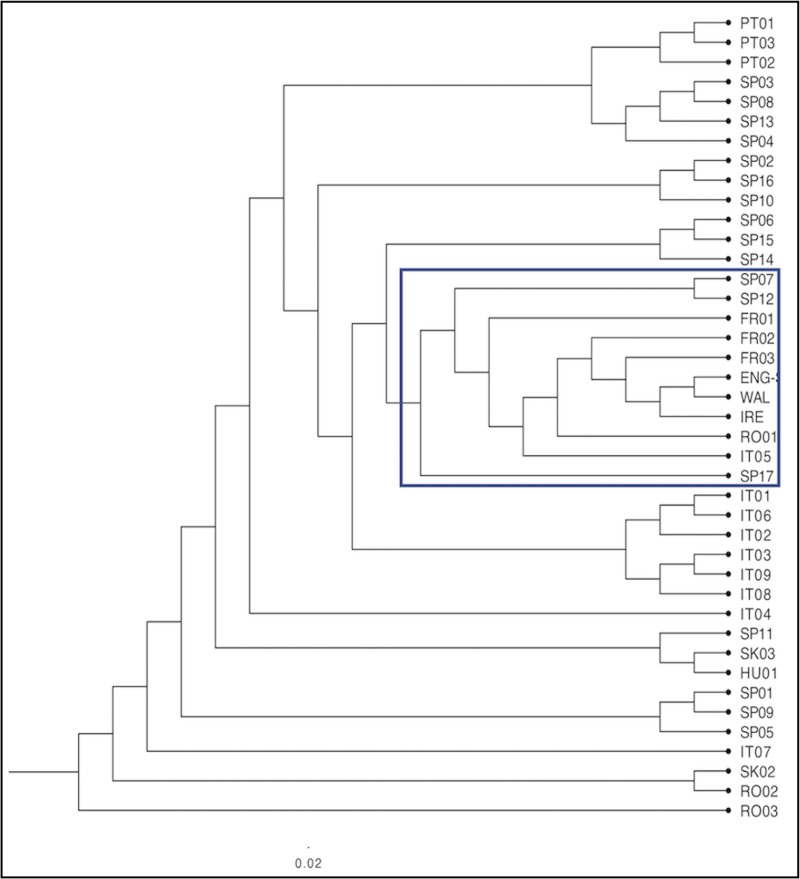
Continental European, England, Ireland and Wales dendrogram. The boxed section is explained in the text.

A dendrogram of the continental European, England, Ireland and Wales sites was produced using UPGMA analysis (F_ST_ Corrected option) (Fig 9). The England (‘ENG’), Ireland (‘IRE’) and Wales (‘WAL’) groups were most closely associated with FR01, FR02, FR03, RO01, IT05, SP17, SP07 and SP12.

**Fig 10:**
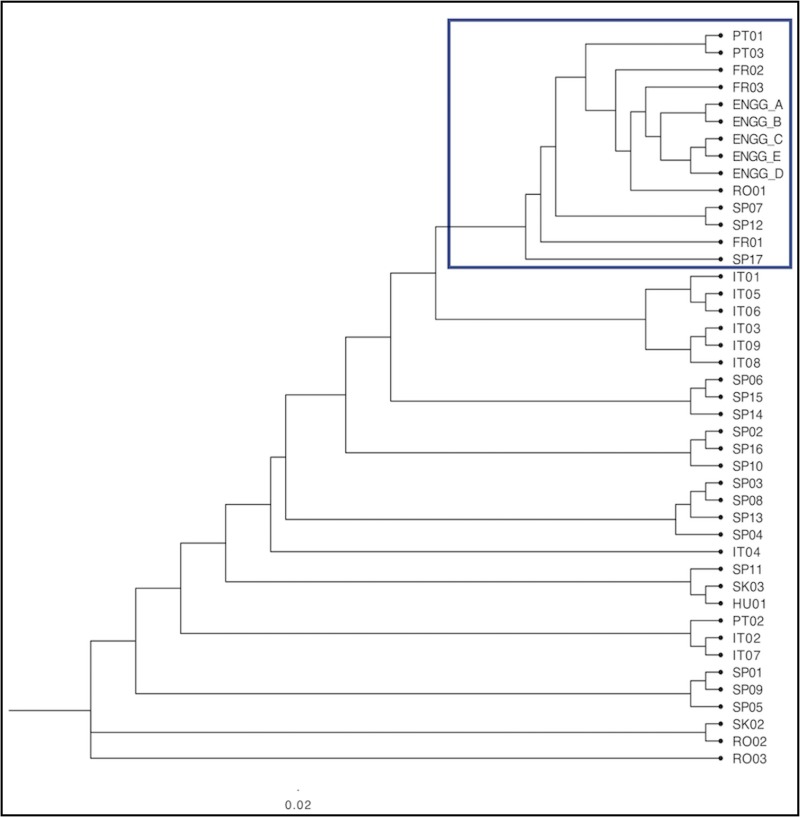
Continental European, England, Ireland and Wales dendrogram, with England, Ireland and Wales samples grouped by Site Types A–E (‘ENGG_A’–‘ENGG_E’). The boxed section is explained in the text.

For comparison, the England, Ireland and Wales samples were re-grouped according to their Site Types A–E (above; S3 File) to test whether older generation trees might derive from different parts of Europe cf. younger generation trees. The resulting dendrogram (Fig 10) indicates broadly the same clustering as Fig 9, but with PT01 and PT03 aligned closer to England, Ireland and Wales; and SP03, SP08, SP13 and SP04 separated from them. The separation of Site Types A and B (younger trees) from Site Types C, D and E (older trees) corroborated the British and Irish data results, above, as summarised in Table 2.

### The PCoA assessment

**Fig 11:**
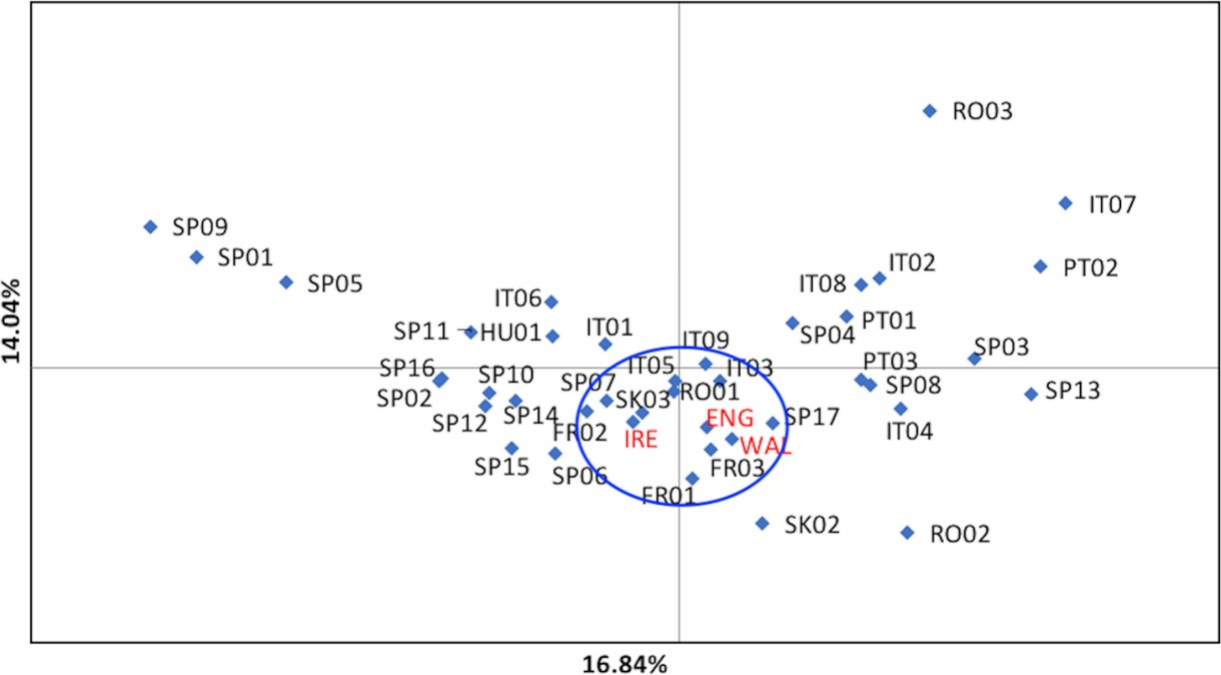
PCoA of continental European, England, Ireland and Wales dataset. Ringed selection indicates England and Wales, offset from Ireland, with associated sites.

PCoA of the continental European, England, Ireland and Wales samples produced Fig 11: England and Wales straddled the centre of the plot (separated from Ireland), most closely associated with FR01, FR03, SP07, SP17, SK03, RO01, IT03, IT05 (similar to the STRUCTURE and UPGMA results). The percentage of variation explained by either axis was relatively low but the outlying positions of RO02, RO03 and SK02 were noteworthy, as these sites appeared similarly anomalous in the F_ST_ and UPGMA analyses.

### Relationships within and between European sites

*F* statistics (F_IS_ and F_ST_) were used to test for relatedness. For the F_IS_ estimation, 38 continental European sites were compared with 143 England, Ireland and Wales sites (excluding single-sample sites): values of F_IS_ ranged from –0.8333 to 0.4783, indicating a wide range presumed caused by diverse numbers of samples per site. (S8 Table provides detailed site values). The pairwise F_ST_ estimation compared 38 continental European sites with the ‘England’, ‘Ireland’ and ‘Wales’ groups and with selected site groups. (S9 Table provides detailed site values). The 10percentile and 90percentile points calculated for each dataset (S10 Table) were found compliant with thresholds for F_ST_ <0.05 (= ‘low’) and >0.15 (= ‘high’) determined in previous studies [8, 10, 58, 59]. Considering samples with pairwise F_ST_ <0.05, a consistent pattern of low differentiation was identified between specific continental European and England, Ireland and Wales sites (S9 Table)–summarised in Table 2. Continental European sites having the closest relatedness with England, Ireland and Wales sites were FR02 and FR03, followed by FR01, RO01, SP12 and PT03; those with consistently high differentiation from England, Ireland and Wales sites were RO03, SK02, IT07, SP01, SP09 and PT02. The PT02 (Portugal) site provides a useful illustration: it was highly differentiated (>0.15) from all British and Irish sites, apart from Silwood (0.035) and Nashdom (0.045), which linked <0.05 with only three other European sites, so their specific linkage with PT02 appears meaningful. Some British and Irish sites displayed no evident connection with any sites: the Tortworth Chestnut linked closest with RO01 (0.1026) but with no other sites <0.148. Three Sisters Bachymbyd only linked with IT08 (0.0396); Bushy House had only one link <0.2, with RO02 (for which *r* analysis yielded LRM 0.204 and QGM 0.720, indicating a weak full-sib/parent-offspring relationship).

The estimation of relatedness using LRM and QGM, using LRM >0.400 and QGM >0.750 to determine strong relationships [60] (S11 Table), showed high relatedness between continental European and England, Ireland and Wales sites in 60 sample pairs for LRM and in 77 pairs for QGM. Reducing the threshold of LRM to >0.25 (a conventional ‘parent–offspring/full sib’ class) yielded 6,841 paired matches within the continental European, British and Irish dataset, indicating a broad network of related samples but focussed on specific sites/regions. Interpreting relatedness from the LRM and QGM methods using microsatellite data requires caution [60], because genotypic similarity can vary according to allele frequencies of a population; however, LRM and QGM estimators are informative of relative kinship among individuals and have been endorsed as robust [61, 62, 63].

Clonal analysis determined clonal relations between and within sites and defined individual tree or stool ‘genotypic size’, supporting the Site Type analyses by determining tree or stool antiquity with an arbitrated girth measurement (below). Clonal matches identified in the England, Ireland and Wales 8-SSR dataset were regarded as superior to any matches identified in the 6-SSR dataset, which were rejected. No clonal matches were found with the continental European dataset, but 56 samples formed 28 pairs of ‘near matches’, of which three differed by a single allele at a single locus so could be presumed somatic mutations, defining three intra-site clonal pairs (within SP13, IT04 and SK03). Six of the ‘near matches’ were between continental European and England sites, but all were >1 allele different, so were not deemed clonal, although close relatedness was evident (S12 Table).

### Site Characterisation groupings of British and Irish sites compared with continental European sites

Three parameter groups from the England, Ireland and Wales study–‘Counties’, ‘Seed Zones’ and ‘Site Types’–were selected as representative of geographical, ecological and historical influences respectively and compared with the continental European sites using F_ST_ analysis (S9 Table; a summary of the results is presented in Table 2).

The ‘Site Types’ grouping yielded PT03, FR03 and RO01 with the highest relatedness with all Site Types at the F_ST_ <0.05 level. Site Type B (modern coppice) sites linked exclusively with IT05 and so appear to originate from continental European sites different from those of ‘ancient coppice’ (Type C) or ‘ancient trees’ (Type D). Another noteworthy finding was that only Type E (ancient garden trees) sites in Britain linked with PT01 (Table 2). For the ‘Seed Zones’ group, there were consistent links between all Seed Zones and FR03 and RO01; and slightly weaker links with PT03, SP12, FR01 and FR02. Seed Zones 304 and 305 (Wales) aligned closest with sites SP06, SP07, SP14 and IT03, with which Ireland did not align (reinforcing the differentiation between Wales and Ireland sites). The ‘Counties’ group displayed a similar pattern: FR03 was the predominant link for all Counties (except Cork and Tipperary, in Ireland), and FR02, RO01, SP07, SP12 and PT03 formed consistent links with most Counties. Sites RO03 and SK02 were consistently highly differentiated from almost all other sites, with F_ST_ frequently >0.30; whereas FR03 and RO01 were consistently undifferentiated across all parameters with all sites, indicating widespread sharing of their genotypes across western Europe.

### Summary of results indicating dominant relationships between British and Irish sites and continental European sites

**Fig 12:**
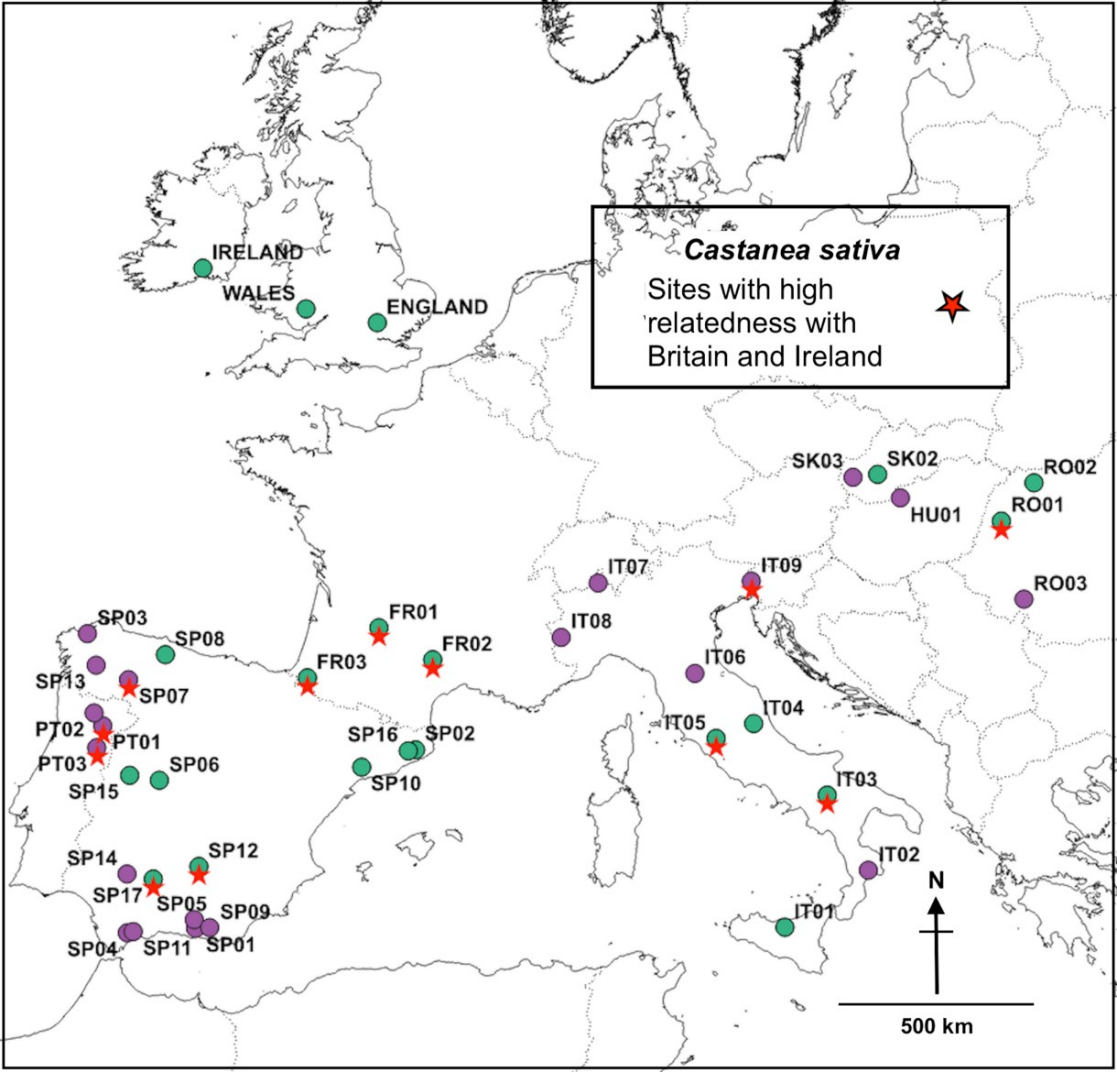
Dominant linkages between England, Ireland and Wales and continental European sites. Map base open-sourced from ‘EuroGeographics and UN-FAO @EuroGeographics’.

In summary, the statistical analyses indicated that sites from England, Ireland and Wales linked predominantly (78%) with ‘Genepool C’ sites in continental Europe; but certain England and Wales sites strongly linked with ‘Genepool D’ (notably with PT03 and SP07) (Table 2). The STRUCTURE map depicts the continental European sites most strongly linked with England, Ireland and Wales–Fig 12: the dominant links are shown with a red star (see S5 File for GIS data).

## Discussion

Discussion will focus on the relationships between *Castanea sativa* genotypes in Britain and Ireland and with those in continental Europe: possible causation for the most likely ancestral populations for British and Irish *C*. *sativa* lying in France (S and SW), Spain (NW and S), Portugal (N), central Italy and Romania will be sought.

### Genotypes of *Castanea sativa* across England, Wales and Ireland

The bipolar genepool identified for Britain and Ireland (Fig 2 and Fig 3), possessing moderate genetic diversity, low levels of inbreeding (F_IS_ = 0.04) and low differentiation between sites (F_ST_ = 0.01), can be explained by analyses of samples grouped by cultural and environmental parameters. The ‘Counties’ analysis (respecting historic cultural divisions within Britain and Ireland) and the ‘Fields of Britannia’ [57] analysis (representing rural landscape regions created in Roman and early medieval periods) consistently differentiated genotypes from Wales, as also revealed by STRUCTURE analysis. The ‘Seed Zones’ [56] analysis (respecting phenotypic cues for tree adaptation) revealed a very weak influence on genotype distribution–as was found in a study of *Fraxinus excelsior* in Britain [64], which mapped haplotype distribution using the same Seed Zones. We suggest that *C*. *sativa* genotypes in Britain and Ireland may not have undergone sufficient reproductive cycles to respond to phenotypic cues, given that even the oldest trees and stools (> *c*. 500 years) were found to be single-generation plants: intra-site recruitment of multiple generations of trees from seed was very rarely observed, to be expected as coppice management typically used layering (vegetative reproduction) to establish new stools [65].

In that context, the genetic differentiation found between ‘younger’ and ‘older’ trees and stools in Britain and Ireland (viz. the difference between Site Types A, B and C, D) apparently indicates a preserved pattern of the origins and chronologies of *C*. *sativa* arrivals from continental Europe. The age differences between the trees and stools in these Site Types were confirmed by the clonal and relatedness analyses, whereby the true dimensions of a tree or stool could be defined genetically rather than visually, providing an accurate ‘antiquity’ measure. The genetic differentiation that was confirmed between ‘modern’ trees in woods and ‘ancient’ trees in parks and historic gardens could be linked with historical nut cultivation: ancient trees renowned for their eating nut qualities were recorded in the historic parks and gardens, indicating historical varietal selection for nuts. Systematic nut production was evinced from archive searches for several sites for the 12^th^–15^th^ centuries AD [1]; and promotion of *C*. *sativa* for nut production in England is recorded from the 17^th^ century AD [28].

Genotypic clusters were revealed in the genetic distance dendrogram (Fig 4), representing inter- and intra-site associations of closely related samples. For example, the main ‘Group A’ clade contained a discrete cluster of samples from the Forest of Dean (Gloucestershire) area known as ‘Castiard’ whence derived some of the earliest written records for *C*. *sativa* in Britain, *c*. AD 1140 [1]. Similarly, the Lydney Park (Gloucestershire) ancient trees formed another cluster, which also included a single tree growing in Ireland. Such clusters could indicate that a narrow range of genotypes had been propagated within a small area and become isolated; or that genotypes were exported from one site to another (as seed and/or vegetatively); or that disjunct sites share close kinship with a common (perhaps geographically remote) ancestor. These findings are similar to those reported for domestication of cultivars of ancient *C*. *sativa* trees in Italy and Iberia [66]; and comparable with a study of the genetic diversity and domestication of *Olea europaea* ssp. *europaea* in the Mediterranean basin [67].

Some single *C*. *sativa* trees were very highly differentiated from all others across Britain and Europe, such as the Tortworth, Seven Sisters Penshurst and Bushy House trees. These ancient ‘single generation’, ‘single clone’ trees are evidently genetically unchanged since their first establishment, so their present ‘difference’ represents the disjunct source(s) from which they originated (and which are excluded from the European sites accessed in this study), rather than evolution of *in situ* genetic isolation.

### Clonal behaviour of *Castanea sativa* defining tree antiquity

A key outcome from this research has been the novel identification of clonal groups in ancient *C*. *sativa* trees and coppice stools: some stools that had been described as ‘massive’ and therefore ‘ancient’ [23] were found to comprise a single genotype, so confirming their great size and antiquity; others were found to be a composite of genotypes so not a massive, necessarily ancient, plant. The clonal analysis has evinced the natural collapse and regeneration of single trees and the historic management of trees and coppice using grafted boughs or layered stems; it also revealed the translocation of vegetative material to establish new plants. Some trees and stools that had appeared ‘clonal’ owing to their growth form were found to be non-clonal, demonstrating the unreliability of visual observation: a tree or stool cannot be considered a ‘single plant structure’ without genetic analysis. The clonal analyses underlined the importance of checking the PCR outputs of the automated binning process using knowledge of the sample’s site context.

The clonal behaviour of *C*. *sativa* recorded in this study was compared with that of other tree species, to verify methods and conclusions. *Prunus avium* [68] and *Populus tremula* [69] can propagate vegetatively by root suckering, with a single clone sometimes occupying thousands of square metres; and *Tilia cordata* [70], *Corylus avellana* [4] and *Quercus pyrenaica* [71] can self-propagate from collapsed boughs and/or root budding. *C*. *sativa* evidently shares the natural habit of *Tilia cordata* of collapsing and regenerating from rooted layers, establishing large clonal patches in the process: clonal growth by bough layering and by stool expansion were observed in the present study for both species across Britain and continental Europe. A study of *Quercus pyrenaica* [71] mapped clonal groups, each occupying many tens of square metres, and found high genetic diversity preserved alongside high clonality, indicating seed regeneration as well as vegetative reproduction: this parallels a study of *C*. *sativa* in Greece [72].

Assessment of *Populus tremula* clones [69] revealed that a very long period (>2000 years) of vegetative regeneration from suckers was accompanied by somatic mutations and a reduction of pollen viability. This is comparable with some ancient *C*. *sativa* trees and stools in the present study, which were evidently single generation plants >500 years old with somatic mutations recorded in some of their vegetatively reproduced components. For some of these sites there may have been only a few *C*. *sativa* trees, even just one during hundreds of years, many kilometres distant from the next. The pollen of *C*. *sativa* is generally locally dispersed [73, 74], although it can be wind-blown long distances [2], so genetic isolation might be anticipated for such remote trees. Interestingly, the only small-island population of *C*. *sativa* sampled within Britain and Ireland in this study (from the Isle of Wight) reported the highest inbreeding coefficient (F_IS_ 0.215).

### The European *Castanea sativa* genepool

The analysis of the continental European, England, Ireland and Wales dataset indicated an overall genepool with high diversity, moderate differentiation and low degree of isolation and inbreeding, in which the England, Ireland and Wales sites were amongst the most diverse. AMOVA results were different from, but comparable with, those from the previous analysis [11] of the European genepool, presumably reflecting the expanded contribution from British and Irish samples.

Two main ‘western’ European genepools were identified: England, Ireland and Wales samples fitted predominantly into ‘Genepool C’, including the ‘oldest’ ancient woodlands and iconic ancient trees; whereas ‘Genepool D’ contained <25% of the England, Ireland and Wales samples, but principally from Wales and historic garden sites.

The assignment by STRUCTURE of sites to ‘Genepool C’ or ‘Genepool D’ was corroborated by the other statistical analyses, except that F_ST_ analysis indicated a strong affiliation between N Portugal (PT03 and PT01) and some England, Ireland and Wales sites. Previous research [11] had assessed Portugal, France, England and Italy as a single ‘Sub-cluster 2’. In considering the potential source regions for British and Irish *C*. *sativa* genepools, N Portugal is an important LGM refugial zone [2] that also shared many cultural connections with Britain, from the Atlantic Bronze Age [75] through to the 18^th^ century AD, when Portuguese sweet chestnuts were especially recommended for propagation [28], perhaps providing linkages between British sites and PT01 and PT03.

The map of final outcomes from the various genetic statistical comparisons (Fig 12) illustrates the importance of the Iberian, Pyrenean and Italian LGM refugial areas as potential sources for the British and Irish *C*. *sativa* genotypes.

### Post-LGM European distribution of *Castanea sativa* compared with other tree species

Previous studies [2, 12, 13] evinced that *C*. *sativa* behaved like other tree species in continental Europe during and post-LGM: it survived in discrete refugia and, following climatic amelioration, spread according to natural and anthropogenic factors [7, 10, 11, 14].

The present study can be compared with phylogenetic studies of other tree species that colonized Britain and Ireland from LGM refugia in continental Europe: *Fraxinus excelsior* [64, 76]; *Quercus robur/Q*. *petraea* [3, 5, 6]; *Tilia cordata* [77]; *Corylus avellana* [4, 78]; and *Fagus sylvatica* [79, 80]. These studies mapped the genotypes/haplotypes of these species across Europe and considered how natural dispersal from LGM refugia and/or anthropogenic translocations contributed to their present distribution: they appear comparable with the spread of *Castanea sativa* from continental Europe to Britain and Ireland.

Studies of *Fraxinus excelsior* in Europe (including Britain) [66, 76] described 12 haplotypes covering Europe: only one was found in Britain and Ireland, which occurs elsewhere only in Iberia, so was concluded to have spread from Iberian LGM refugia to Britain and Ireland along the Atlantic seaboard. AMOVA and STRUCTURE statistics described a single, genetically diverse population covering most of Britain; F_ST_ values between Britain and France were very low. This work on *F*. *excelsior* closely parallels the results of the present study for *C*. *sativa*.

*Corylus avellana* genotypes have been mapped across western Europe [4, 78], indicating a rapid post-LGM expansion from a refugium in the Biscay area into western Europe, Britain and Ireland, presumed to have been facilitated by nut-eating birds and mammals (jays, nuthatches, squirrels) with repeated long-distance dispersal of nuts. Genetic, historical and archaeological data indicated that hazelnuts were a food source for early people and *C*. *avellana* may have been deliberately or accidentally spread [4], potentially similar to *Castanea sativa*.

Studies of *Quercus robur* and *Q*. *petraea* in Britain and Ireland [3, 5, 6] indicated three predominant haplotypes (see Fig 1 and Fig 2 in [5]) that originated in a LGM refugium in west Iberia and migrated north and west into Britain and Ireland along the Atlantic seaboard. These studies considered the rates of post-LGM migration necessary before Irish Sea and English Channel land bridges flooded and stated that long-distance dispersal was required, by ‘birds and rare climatic storms’ [6]. The role of anthropogenic translocation was also considered (see Fig 1A and Fig 1B in [3]): two routes were proposed–the ‘Atlantic lineage’ spread from Spain to Scandinavia; and the ‘Balkan lineage’ spread from east to west Europe, north of the Alps. Post-LGM migration routes for hominids and for oak appeared very similar, such that anthropogenic translocation might explain ‘islands’ of oak genotypes where one haplotype has evidently ‘leapfrogged’ another, as found in France and in Britain [3]. Acorns were an important food for humans and their livestock and varietal selection and propagation appear to have influenced oak genotype distribution [3]: parallels with *C*. *sativa* are evident.

The distribution of *Fagus sylvatica* across Europe has been assessed using palaeobotanical and genetic evidence [79, 80]: post-LGM colonization was from mountain refugia in N Spain and SW France to western continental Europe; and from refugia in E Alps-Slovenia-Istria, skirting north of the Alps to France and thence to England. *F*. *sylvatica* genotypes in Britain [80] show a broad admixture, with regional differentiation inherited from several continental European sources: patterns formed by natural colonization were found to persist despite anthropogenic interventions. *F*. *sylvatica* nuts were an important food for humans and their livestock, as were *C*. *sativa* nuts [81].

A study of LGM refugia in the Pyrenees and northwest Iberia [82] indicated that many tree species (including *C*. *sativa*) survived there and that from 6000 yr BP deciduous broadleaved trees spread to colonize northern Iberia: refugia for *C*. *sativa* have been corroborated on the Cantabrian coast and in S Galicia and N Portugal [8, 12]. These Pyrenean and Iberian refugia appear to be the predominant regional sources of the *C*. *sativa* genotypes found by the present study in England, Ireland and Wales.

The expansion of *C*. *sativa* from LGM refugia in Iberia and S and W France [2, 13], evidently has similarities with the expansion from refugia of *Q*. *robur*/*Q*. *petraea*, *F*. *sylvatica*, *F*. *excelsior* and *C*. *avellana*. The ecological niche of *C*. *sativa* is broadly similar to *Q*. *robur*/*Q*. *petraea*, *F*. *sylvatica* and *C*. *avellana*, in terms of soil and climate preferences and seed (nut) dispersal. Crucially, *C*. *sativa* has long been favoured by humans for food, and anthropogenic interventions in its dispersal and varietal selection have synergised with natural processes [14], possibly paralleling the natural/anthropogenic spread of *Q*. *robur*/*Q*. *petraea*, *F*. *sylvatica* and *C*. *avellana*. However, there is no evidence for natural spread of *C*. *sativa* to Britain and Ireland.

## Conclusions

Analysis of the genetic diversity of *Castanea sativa* in England, Ireland and Wales revealed a single overall genepool, with two sub-clusters: Wales sites, differentiated from Ireland. The England, Ireland and Wales sites had their strongest European connections with sites in south and west France, northern Iberia, central Italy, and a site in Romania: no connections were found with the eastern European genepools in Greece, the Balkans and Turkey. The relatively high diversity of the England, Ireland and Wales *C*. *sativa* genepool is considered the product of several arrivals of seed and/or living plant material from these European zones. Sites in Britain and Ireland with ancient trees and coppice stools (Types C and D) were genetically differentiated from sites with relatively recent (<200 years age) trees and stools (Types A and B), indicating ‘modern’ selection of seed/rooted plants from new sources and not from already established stands. Sites in Britain with ancient historic garden trees (Type E) were strongly affiliated with sites in N Portugal and NW Spain.

Clonal analysis of British and Irish samples determined for the first time the ‘genotypic size’ of ancient trees and stools and thereby their great antiquity, including the largest *C*. *sativa* stools and ancient trees recorded in Britain. Clonal evidence revealed both natural regeneration and anthropogenic propagation of trees and coppice, through bough collapse, layering and planting of vegetative material. Historical systematic nut production was evinced for several sites, where extant ancient trees renowned for their eating nut qualities indicate past selection for nuts, including two examples of ancient trees with grafted boughs.

The ancient *C*. *sativa* trees and coppice stools in Britain are considered single-generation survivors of the original planted or self-sown trees. Genetic stability of *C*. *sativa* was evinced from the clonal studies, sustained through repeated cutting, demise and regrowth over many centuries: a few somatic mutations were recorded.

The conservation significance of ancient *C*. *sativa* trees and stools in Britain and Ireland has been highlighted by this study: their genetic diversity is important to considerations of future risks from pathogens and environmental change. The Future Trees Trust *Castanea sativa* collection was shown to consist of a broad genetic base representative of the overall British and Irish genepool.

## Supporting information

S1 File Sample locations England, Ireland and Wales .kml file. (KML)

S2 File ‘Genepool A’ and ‘Genepool B’ sample locations England, Ireland and Wales .kml file. (KML)

S3 File Results of five parameters analyses England, Ireland and Wales. (DOCX)

S4 File STRUCTURE analysis European including England, Ireland and Wales samples. (DOCX)

S5 File ‘Genepool C’ and ‘Genepool D’ sample locations Europe including England, Ireland and Wales .kml file. (KML)

S1 Fig Dendrogram of England, Ireland and Wales samples using Nei genetic distance. (PDF)

S1 Table Database for England, Ireland and Wales samples with site information. (XLSX)

S2 Table AMOVA England, Ireland and Wales samples. (DOCX)

S3 Table Results of F_ST_ analysis for England, Ireland and Wales samples and site parameter distributions. (XLSX)

S4 Table Results of *r* relatedness analysis for England, Ireland and Wales samples using LRM and QGM. (XLSX)

S5 Table LRM and QGM results for ‘Castiard’ study site. (XLSX)

S6 Table Database European including England, Ireland and Wales samples. (XLSX)

S7 Table Genetic diversity analysis European including England, Ireland and Wales samples. (DOCX)

S8 Table Results of F_IS_ analysis of European including England, Ireland and Wales samples. (XLSX)

S9 Table Results of F_ST_ analysis of European including England, Ireland and Wales samples. (XLSX)

S10 Table F_ST_ threshold values. (DOCX)

S11 Table Results of *r* relatedness analysis for European including England, Ireland and Wales samples using LRM and QGM. (XLSX)

S12 Table Results of clonal analysis of European including England, Ireland and Wales samples. (XLSX)
