# Supplementary material for: DNA analysis of Castanea sativa (sweet chestnut) in Britain and Ireland: Elucidating European origins and genepool diversity
Source: PLoS One. 2019 Sep 25;14(9):e0222936. doi: 10.1371/journal.pone.0222936 (PMC6760806; doi:10.1371/journal.pone.0222936)
Supplement: S3 File — (DOCX) [file pone.0222936.s003.docx]

**S3 File. Site Characterisation parameters for British and Irish samples**

1. Map Grid – 100km Grid squares. All the samples were sorted by their respective 100 km grid square location, covering Britain and Ireland (S3 Fig 1): the samples occupied 27 discrete grid squares.


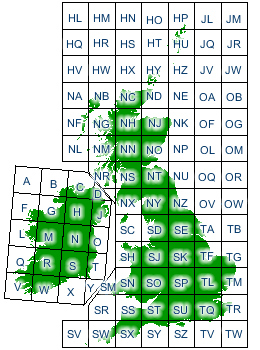


S3 Fig 1. 100km grid squares defined by the British and Irish mapping systems.

The map grid is a convenient structure for presenting the samples within a north–east–south–west distribution independent of cultural or environmental boundaries. Grid squares with <5 samples were excluded (incl. Scotland and N England) and the Ireland sites were amalgamated into a single group ‘IRE’: 15 grid squares (containing 604 ex 611 samples) were assessed. PCoA analysis indicated 3 groups (ringed in S3 Fig 2): ‘TL’ and ‘TR’; ‘SJ’ and ‘SK’; ‘IRE’; and the rest.

S3 Fig 2**.** PCoA England Ireland Wales sampled allocated by 100km Map Grid. The ringed groups are explained in the text.

F_IS_ values ranged from -0.074 for ‘TR’ to 0.145 for ‘SW’, indicating a low–moderate level of isolation and inbreeding; ‘TL’ (F_IS_ 0.007) and ‘SK’ (F_IS_ 0.092) were at opposite ends of the spectrum indicated by the PCoA. The F_ST_ pairwise estimation indicated no differentiation >0.05 between groups: the greatest difference was between ‘TL’ and ‘SK’ with F_ST_ =0.033, replicating the differentiation shown in the PCoA. The 90%ile threshold was 0.015.

2. Administrative Counties and Regions of Britain and Ireland. The 611 samples were sorted by their County location: 44 counties contained samples, but counties with <5 samples were discarded, leaving 25 counties (577 samples) for analysis (minus Scotland and N England). The county boundaries respect historical divisions within Britain and Ireland, based on geographical and cultural boundaries.

Three recognisable County clusters were evident from the PCoA Chart (S3 Fig 3a), which were replicated in the UPGMA dendrogram (S3 Fig 3b): Monmouthshire, Caerphilly, Herefordshire and Kerry; W Sussex, E Sussex and Hampshire; and a group straddling south western, central and eastern England and Powys (Wales).

S3 Fig 3a. PCoA, England Ireland Wales, 25 Counties, ringed groups explained in the text.

F_IS_ values ranged from -0.057 for Somerset to 0.118 for Shropshire. The F_ST_ pairwise estimates showed the majority of County groups with no differentiation >0.05: four Counties had scores >0.05 up to 0.092; the 90%ile threshold was 0.044.

S3 Fig 3b. UPGMA F_ST_ Corr. Dendrogram, England Ireland Wales, 25 Counties, coloured groups explained in the text.

The British and Irish dataset was sorted into the administrative Regions of England (the former Government Office regions) and the countries of Scotland, Wales and Ireland: those with <5 samples – NW England, Yorkshire, Scotland –were excluded, providing six regions for analysis. PCoA presented a spatial separation (S3 Fig 4a) that was replicated in the UPGMA analysis (S3 Fig 4b).

S3 Fig 4a. PCoA England, Ireland, Wales, Regions and countries distribution of samples.

S3 Fig 4b. UPGMA F_ST_ Corr. Dendrogram, England, Ireland and Wales, Regions/countries distribution of samples.

F_IS_ values ranged from 0.012 for WM to 0.08 for WAL, with IRE at 0.066. F_ST_ pairwise estimates provided no regions >0.05; Wales and Ireland were the most differentiated regions, at 0.014, replicating the pattern in the PCoA and UPGMA charts. The 90%ile threshold was 0.010.

3. Seed Zones. Fourteen forest seed zones (S3 Fig 5a) represent the samples in Britain; Ireland is covered by a single seed zone. Seed zones with <5 samples were excluded from the analyses, leaving 9 seed zones with 603 samples (excluding Scotland, N England).

S3 Fig 5a. Seed Zones for Britain (Ireland is a single Seed Zone, not mapped) [85].


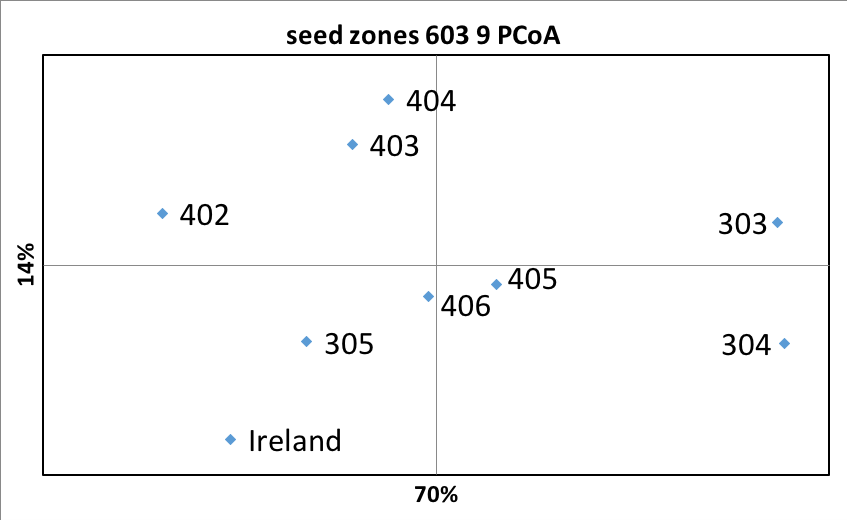


S3 Fig 5b. PCoA England, Ireland, Wales, Seed Zones. The ringed groups are explained in the text.

The PCoA chart shows Zones 303 and 304 (covering Wales) at one extreme of the *x*-axis ( at 70%), separated from Zone 402 (E Midlands and central England) and Ireland at the other. The central cluster represents southern England and E Anglia.

F_IS_ values ranged from -0.03 for 305 to 0.098 for 304 and 0.099 for 402. The F_ST_ pairwise estimates provided no seed zones with values >0.05; the largest score was 0.021, between Ireland and Zones 303 and 304 (Wales) – replicating the pattern shown in the PCoA chart (S3 Fig 5b). The 90%ile threshold was 0.017.

4. ‘Fields of Britannia’ regions. The England and Wales samples were sorted according to the historical landscape regions devised by the ‘Fields of Britannia’ project [86] – S3 Fig 6a. The ‘Northern Uplands’ region was disregarded (only one sample).


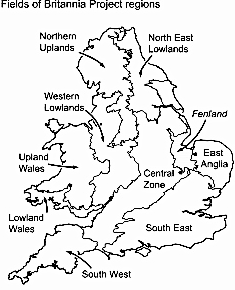


S3 Fig 6a. ‘Fields of Britannia’ regions for England and Wales [86].

S3 Fig 6b. PCoA, ‘Fields of Britannia’ regions, England and Wales. Separation of groups explained in the text.

The PCoA analysis *x*-axis (72.5%) indicates significant separation between Central Zone and Lowland Wales/Upland Wales, with a central cluster of the other regions.

F_IS_ values ranged from -0.006 for South West to 0.09 for Central Zone. There were no F_ST_ pairwise estimate scores >0.05; the greatest differentiation was between Central Zone and Lowland Wales and Upland Wales – as demonstrated in the PCoA chart (S3 Fig 6b). The 90%ile threshold was 0.015.

5. Site Type. The survey data enabled the categorisation of all the sites in Britain and Ireland into five main types.

A High forest woodland with single stemmed (standard) *Castanea sativa* trees, modern origin <~200 years

B Coppice woodland with small *Castanea sativa* stools, modern origin <~200 years

C Ancient *Castanea sativa* coppice stools, typically in designated ancient semi-natural woodland (wooded since at least AD 1600), within high forest or coppice woodland

D Ancient *Castanea sativa* trees in parkland (historic and/or designed landscape of pasture with scattered veteran trees and groups of trees); ancient tree, stub or stool in a hedgerow or on a field bank (medieval boundaries, historic agricultural features and/or relic of ancient woodland clearance)

E Ancient *Castanea sativa* trees (often single specimens) in gardens and grounds in close proximity to historic houses (that may no longer be extant).

The England, Ireland and Wales samples were sorted according to these five Site Types. The PCoA chart (S3 Fig 7) showed a clear separation on the x-axis of Type B (modern coppice) from Type C (ancient coppice) and the other veteran tree categories (D & E). Type A represents standard trees of various ages in high forest and straddles the ‘old versus new’ spectrum.

S3 Fig 7: PCoA, Site Types for England, Wales and Ireland.

F_IS_ values ranged from 0.027 for Type D to 0.111 for Type E. The F_ST_ analysis indicated low differentiation (<0.05) between all the Site Types, with the greatest differentiation (F_ST_ 0.010) between Type B and Types C and D – as also demonstrated in the PCoA chart (S3 Fig 7); the 90%ile threshold was 0.011. A postulated geographical influence that might explain this clustering of samples was examined and rejected.
