# Supplementary material for: DNA analysis of Castanea sativa (sweet chestnut) in Britain and Ireland: Elucidating European origins and genepool diversity
Source: PLoS One. 2019 Sep 25;14(9):e0222936. doi: 10.1371/journal.pone.0222936 (PMC6760806; doi:10.1371/journal.pone.0222936)
Supplement: S4 File — (DOCX) [file pone.0222936.s004.docx]

**S4 File. STRUCTURE analysis of the western Eurasian, England, Ireland and Wales sites**

Several permutations of the combined western Eurasia, England, Ireland and Wales dataset were run in STRUCTURE, to test the impact of using 6 SSRs or 5 SSRs (minus EMCs25) and of including or excluding several sites (from BU, GR, RO, SK and TR). The results are presented here.

The full dataset of samples from England, Ireland and Wales were incorporated with the full original dataset of the western Eurasian populations [11], (which had only included the small preliminary samples from west England), to comprise 2199 samples from 319 sites (247 British & Irish, 72 Eurasian), using 6 SSRs (S4 File Fig 1a-b).


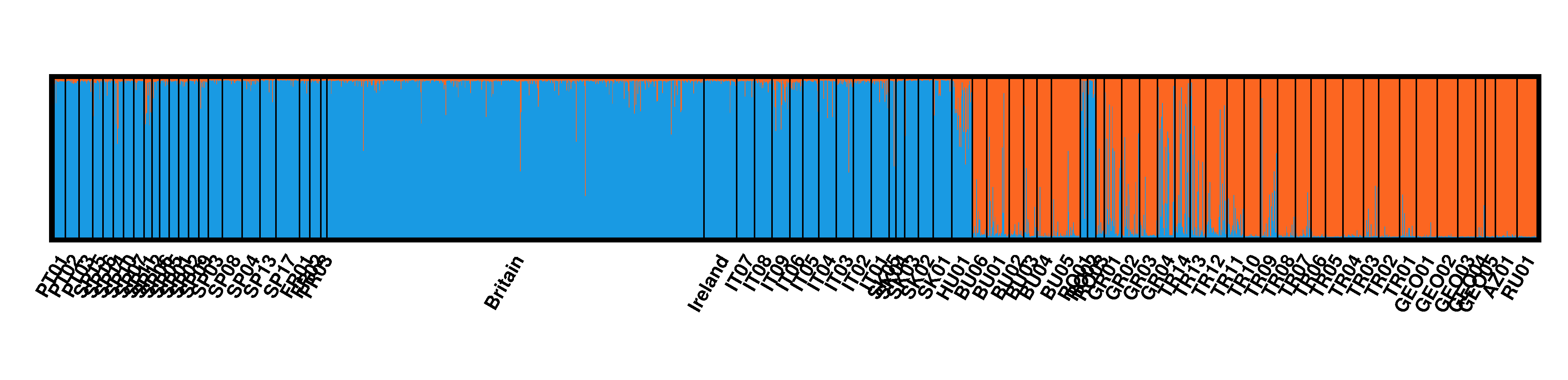


S4 File Fig 1a. STRUCTURE output Series 1 and Series 2, 6 SSRs, 2199 samples, 74 groups, for all western Eurasian sites (including the full British and Irish dataset here grouped as ‘Britain’ and ‘Ireland’).

S4 File Fig 1b. STRUCTURE output, K=2 Evanno, 6 SSRs, 2199 samples, 74 groups, for all western Eurasian sites (including the full British and Irish dataset here grouped as ‘Britain’ and ‘Ireland’).

For comparison, the original STRUCTURE output for the western Eurasian data with the smaller England dataset is reproduced (S4 File Fig 1c), with K=2 with a subpopulation of K’=3.


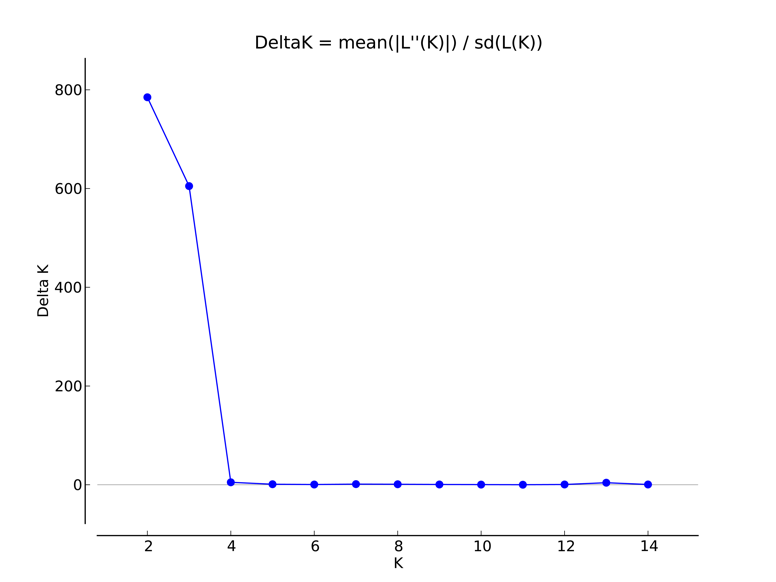


S4 File Fig 1c. The deltaK chart [11] (K=2 Evanno with a subpopulation of K’=3) produced for the western Eurasian dataset with the smaller England dataset

The STRUCTURE analysis (S4 File Fig 1a) indicated a clear separation among the eastern and western Eurasian populations, evaluated by CLUMP as two populations (*K*=2 Evanno) with a minor peak at K=5 (S4 File Fig 1b). The eastern (orange) and western (blue) populations of western Eurasia are clearly differentiated (S4 File Fig 1a), replicating the previous findings [11] even with the full set of additional British and Irish samples. This analysis indicated no matching of the British or Irish samples with any of the eastern European populations, except marginally with Bulgaria (BU01), Greece (GR04) and Turkey (TR14).

A subset of the predominantly ‘blue’ sites in S4 File Fig 1a was selected for further analysis, covering all the British and Irish sites, France, Hungary, Italy, Portugal, Romania, Slovakia and Spain, with BU01, GR04 and TR14. This dataset contained 1430 samples, from 44 groups of sites from 13 countries. STRUCTURE analysis indicated the most likely number of populations within the dataset as five (*K*=5 Evanno) (S4 File Figs 2a-c). The *ΔK* score of 25 at the *K*=5 peak is very low (and perhaps reflects the minor peak for *K*=5 in S4 File Fig 1b).

S4 File Fig 2a. STRUCTURE output, Series 1–Series 5, 44 western Eurasian groups including England (ENG-SCO), Wales (WAL) and Ireland (IRE). 6 SSRs, 1430 samples.

S4 File Fig 2b. STRUCTURE output, *K*=5 Evanno, 44 western Eurasian groups including England, Wales and Ireland, 1430 samples.

S4 File Table 1. STRUCTURE HARVESTER output, western Eurasia, England, Wales and Ireland, 6 SSRs, 1430 samples, 44 groups.

| **K** | **Reps** | **Mean LnP(K)** | **Stdev LnP(K)** | **Ln'(K)** | **\|Ln''(K)\|** | **Delta K** |
| --- | --- | --- | --- | --- | --- | --- |
| 1 | 6 | -35364.616667 | 0.098319 | — | — | — |
| 2 | 6 | -34032.133333 | 148.075436 | 1332.483333 | 203.600000 | 1.374975 |
| 3 | 6 | -32903.250000 | 238.235100 | 1128.883333 | 489.283333 | 2.053784 |
| 4 | 6 | -32263.650000 | 42.153802 | 639.600000 | 47.400000 | 1.124454 |
| 5 | 6 | -31671.450000 | 12.077044 | 592.200000 | 299.033333 | **24.760473** |
| 6 | 6 | -31378.283333 | 15.340328 | 293.166667 | 71.716667 | 4.675041 |
| 7 | 6 | -31013.400000 | 16.687600 | 364.883333 | 143.933333 | 8.625167 |
| 8 | 6 | -30792.450000 | 32.725082 | 220.950000 | 10.450000 | 0.319327 |
| 9 | 6 | -30581.950000 | 49.367388 | 210.500000 | 318.166667 | 6.444875 |
| 10 | 6 | -30689.616667 | 204.678796 | -107.666667 | — | — |

The *K*=5 data was used to assign each site to a specific Series, using an arbitrary threshold of *Q*≥0.7 to define the sites with the highest proportion of their samples within each of the five Series (a threshold of *Q*≥0.75 had been used by [10]). The relative contribution of each of the 44 groups to each *K* series is charted in S4 File Fig 2c. Series 5 (pale blue) is dominated by the samples from TR14, GR04 and BU01 and also RO03, which appear outwith the main population group.

S4 File Fig 2c. STRUCTURE output, Series 1-5, western Eurasia, England, Wales and Ireland, 44 groups, 1430 samples.

There were only five samples from Britain and Ireland with Q≥0.7 in Series 5, which did not display any evident inter-relationships, so it was decided to remove BU01, GR04 and TR14 from the dataset. In this analysis it was evident that RO02, RO03 and SK03 were also markedly variable and different, so they were re-evaluated *infra*.

STRUCTURE was run without BU01, GR04 and TR14, for 41 groups (1332 samples) – S4 File Fig 3a. The most likely number of populations within this dataset was assessed as four (*K*=4 Evanno) with a peak value of Δ*K* =38, again relatively low (S4 File Fig 3b and S4 File Table 2).

S4 File Fig 3a. STRUCTURE output, Series 1–Series 4, western Eurasia, England (ENG-SCO), Ireland (IRE) and Wales (WAL), 6 SSRs, 1332 samples, 41 groups (10 countries).

S4 File Fig 3b. STRUCTURE output, K=4 Evanno, western Eurasia, England, Ireland and Wales, 6 SSRs, 1332 samples, 41 groups.

S4 File Table 2. STRUCTURE HARVESTER output, western Eurasia, England, Ireland and Wales, 6 SSRs, 1332 samples, 41 groups.

| **K** | **Reps** | **Mean LnP(K)** | **Stdev LnP(K)** | **Ln'(K)** | **\|Ln''(K)\|** | **Delta K** |
| --- | --- | --- | --- | --- | --- | --- |
| 1 | 6 | -32180.616667 | 0.075277 | — | — | — |
| 2 | 6 | -30785.716667 | 150.343891 | 1394.900000 | 613.950000 | 4.083638 |
| 3 | 6 | -30004.766667 | 192.455456 | 780.950000 | 126.133333 | 0.655390 |
| 4 | 6 | -29349.950000 | 4.481852 | 654.816667 | 306.100000 | **68.297655** |
| 5 | 6 | -29001.233333 | 3.936327 | 348.716667 | 82.166667 | 20.873946 |
| 6 | 6 | -28734.683333 | 8.175431 | 266.550000 | 56.450000 | 6.904835 |
| 7 | 6 | -28524.583333 | 7.348855 | 210.100000 | 44.683333 | 6.080312 |
| 8 | 6 | -28359.166667 | 2.920046 | 165.416667 | — | — |

This K=4 peak was compared with the STRUCTURE analysis of the ‘England Ireland Wales’ dataset that had used 6 SSRs instead of 8 SSRs: the 6-SSR analysis had revealed a strong K=4 peak, whereas the 8-SSR analysis of the same data had revealed only a single, stronger K=2 peak. In that context, the 6-SSR dataset was regarded as less accurate than the 8-SSR dataset and so the K=4 output was discarded. To test the relative effect of the British and Irish data on the Eurasian dataset, the western Eurasian samples (724 from 38 groups) were assessed without the England, Ireland, Wales samples: using 6 SSRs, STRUCTURE produced a strong K=2 peak, with lesser clusters at K=3 and K=4 as shown in S4 File Fig 3.

S4 File Fig 3. STRUCTURE output, K=2 Evanno, western Eurasia without England, Ireland and Wales, 6 SSRs, 724 samples, 38 groups

S4 File Table 3. STRUCTURE HARVESTER output, western Eurasian samples without England, Ireland, Wales, 6 SSRs, 724 samples, 38 groups.

| **K** | **Reps** | **Mean LnP(K)** | **Stdev LnP(K)** | **Ln'(K)** | **\|Ln''(K)\|** | **Delta K** |
| --- | --- | --- | --- | --- | --- | --- |
| 1 | 6 | -17937.000000 | 0.063246 | — | — | — |
| 2 | 6 | -17114.183333 | 0.563619 | 822.816667 | 226.050000 | **401.068624** |
| 3 | 6 | -16517.416667 | 1.485149 | 596.766667 | 182.983333 | 123.208762 |
| 4 | 6 | -16103.633333 | 2.471167 | 413.783333 | 150.100000 | 60.740531 |
| 5 | 6 | -15839.950000 | 59.734705 | 263.683333 | 69.400000 | 1.161804 |
| 6 | 6 | -15506.866667 | 17.688037 | 333.083333 | — | — |

To cross-check the veracity of the analyses further using 6 SSRs, the western Eurasia, England, Ireland and Wales dataset was revised to exclude EMCs25, the locus that presented null alleles. The 5-SSR dataset produced *K*=2 at Δ*K*=172, with a secondary peak K=4 at Δ*K*=105 (S4 File Fig 4, Table 4).

S4 File Fig 4. STRUCTURE output, K=2 Evanno, western Eurasia, England, Ireland and Wales, 5 SSRs, 1329 samples, 41 groups

S4 File Table 4. STRUCTURE HARVESTER output, western Eurasia, England, Ireland and Wales, 5 SSRs (minus EMCs25), 1329 samples, 41 groups.

| **K** | **Reps** | **Mean LnP(K)** | **Stdev LnP(K)** | **Ln'(K)** | **\|Ln''(K)\|** | **Delta K** |
| --- | --- | --- | --- | --- | --- | --- |
| 1 | 6 | -28341.300000 | 0.000000 | — | — | — |
| 2 | 6 | -27585.183333 | 1.799352 | 756.116667 | 308.850000 | **171.645151** |
| 3 | 6 | -27137.916667 | 16.455080 | 447.266667 | 19.183333 | 1.165800 |
| 4 | 6 | -26671.466667 | 3.746287 | 466.450000 | 393.416667 | 105.015089 |
| 5 | 6 | -26598.433333 | 359.382925 | 73.033333 | 160.466667 | 0.446506 |
| 6 | 6 | -26364.933333 | 56.615604 | 233.500000 | 115.116667 | 2.033303 |
| 7 | 6 | -26246.550000 | 98.752595 | 118.383333 | 126.783333 | 1.283848 |
| 8 | 6 | -26001.383333 | 27.314422 | 245.166667 | — | — |

The K=4 output for the combined western Eurasia, England, Ireland, Wales dataset using 6 SSRs (1332 samples, 41 groups) was thereby presumed to be suspect and was disregarded in the further analyses.

A further test was run in STRUCTURE using the western Eurasia, England, Ireland, Wales dataset without RO02, RO03 and SK02, first with 6 SSRs and then with 5 SSRs (excluding EMCs25). The 6-SSR dataset had 1287 samples and STRUCTURE produced K=4 (S4 File Fig 5; Table 5).

S4 File Fig 5. STRUCTURE output, K=4 Evanno, western Eurasia, England, Ireland and Wales, 6 SSRs, 1287 samples, 38 groups (excluding SK02, RO02, RO03)

S4 File Table 5. STRUCTURE HARVESTER output, western Eurasia, England, Ireland and Wales, 6 SSRs, 1287 samples, 38 groups (excluding SK02, RO02, RO03)

| **K** | **Reps** | **Mean LnP(K)** | **Stdev LnP(K)** | **Ln'(K)** | **\|Ln''(K)\|** | **Delta K** |
| --- | --- | --- | --- | --- | --- | --- |
| 1 | 6 | -30960.400000 | 0.000000 | — | — | — |
| 2 | 6 | -29704.900000 | 197.543757 | 1255.500000 | 529.583333 | 2.680841 |
| 3 | 6 | -28978.983333 | 429.465144 | 725.916667 | 5.350000 | 0.012457 |
| 4 | 6 | -28247.716667 | 3.356437 | 731.266667 | 386.600000 | **115.181678** |
| 5 | 6 | -27903.050000 | 19.942091 | 344.666667 | 35.416667 | 1.775976 |
| 6 | 6 | -27522.966667 | 25.784621 | 380.083333 | 304.116667 | 11.794498 |
| 7 | 6 | -27447.000000 | 5.091562 | 75.966667 | 77.050000 | 15.132882 |
| 8 | 6 | -27448.083333 | 18.604775 | -1.083333 | — | — |

The 5-SSR dataset had 1284 samples (38 groups) and STRUCTURE produced K=2 Evanno (S4 File Fig 6; Table 6).

S4 File Fig 6. STRUCTURE output, K=2 Evanno, western Eurasia, England, Ireland and Wales, 5 SSRs (minus EMCs25), 1284 samples, 38 groups (excluding SK02, RO02, RO03)

S4 File Table 6. STRUCTURE HARVESTER output, western Eurasia, England, Ireland and Wales, 5 SSRs (minus EMCs25), 1284 samples, 38 groups (excluding SK02, RO02, RO03)

| **K** | **Reps** | **Mean LnP(K)** | **Stdev LnP(K)** | **Ln'(K)** | **\|Ln''(K)\|** | **Delta K** |
| --- | --- | --- | --- | --- | --- | --- |
| 1 | 6 | -27306.983333 | 0.040825 | — | — | — |
| 2 | 6 | -26576.583333 | 1.416216 | 730.400000 | 268.950000 | **189.907523** |
| 3 | 6 | -26115.133333 | 11.085065 | 461.450000 | 11.833333 | 1.067502 |
| 4 | 6 | -25665.516667 | 2.581020 | 449.616667 | 248.550000 | 96.299120 |
| 5 | 6 | -25464.450000 | 4.548296 | 201.066667 | 639.750000 | 140.657061 |
| 6 | 6 | -25903.133333 | 1326.786064 | -438.683333 | 1137.116667 | 0.857046 |
| 7 | 6 | -25204.700000 | 21.553654 | 698.433333 | 963.650000 | 44.709357 |
| 8 | 6 | -25469.916667 | 539.386027 | -265.216667 | — | — |

Comparison of the K2-pop results for the Eurasian sites in each output showed identical Q values, so it was evident that the England, Ireland, Wales sites using 6 SSRs generated the K=4 result. Given the 8-SSR output for the England, Ireland, Wales sites was K=2, the overall western Eurasia, England, Ireland and Wales population structure was determined as K=2.

To test an alternative configuration of the British and Irish samples, the five Site Type groups A–E for the England Ireland Wales samples were incorporated into the Eurasian dataset and run in STRUCTURE for 6 SSRs, 1332 samples and 43 groups: K=2 Evanno was the result (S4 File Fig 7, Table 7). The Site Types represent a typological grouping of samples, they are not geospatial sites so are not directly comparable with the Eurasian sites.

S4 File Fig 7. STRUCTURE output, K=2 Evanno, western Eurasia, England, Ireland and Wales, 6 SSRs, 1332 samples, 43 groups (England Ireland Wales, 5 Site Types A–E)

S4 File Table 7. STRUCTURE HARVESTER output, western Eurasia, England, Ireland and Wales, 6 SSRs, 1332 samples, 43 groups (England Ireland Wales, 5 Site Types A–E)

| **K** | **Reps** | **Mean LnP(K)** | **Stdev LnP(K)** | **Ln'(K)** | **\|Ln''(K)\|** | **Delta K** |
| --- | --- | --- | --- | --- | --- | --- |
| 1 | 6 | -32180.666667 | 0.051640 | — | — | — |
| 2 | 6 | -30725.333333 | 1.552632 | 1455.333333 | 676.816667 | **435.915652** |
| 3 | 6 | -29946.816667 | 179.861373 | 778.516667 | 178.433333 | 0.992060 |
| 4 | 6 | -29346.733333 | 6.980449 | 600.083333 | 255.466667 | 36.597455 |
| 5 | 6 | -29002.116667 | 5.323689 | 344.616667 | 66.283333 | 12.450639 |
| 6 | 6 | -28723.783333 | 24.127198 | 278.333333 | 87.250000 | 3.616251 |
| 7 | 6 | -28532.700000 | 3.860570 | 191.083333 | 41.633333 | 10.784245 |
| 8 | 6 | -28383.250000 | 21.561702 | 149.450000 | — | — |

Lastly, to provide a deeper breakdown of the England, Ireland and Wales sites representing their geographical distribution and so conforming with the Eurasian sites, the Administrative Counties classes (25 counties in England, Ireland and Wales) were incorporated into the western Eurasian dataset, giving 1294 samples with 63 groups. STRUCTURE analysis yielded K=2 Evanno (S4 File Fig 8a-b, Table 8).

S4 File Fig 8a. STRUCTURE output, K=2 Evanno, western Eurasia, England, Ireland and Wales, 6 SSRs, 1294 samples, 63 groups (25 England Ireland Wales Counties)

S4 File Table 8. STRUCTURE HARVESTER output K=2 Evanno, western Eurasia, England, Ireland and Wales, 6 SSRs, 1294 samples, 63 groups (25 England Ireland Wales Counties)

| **K** | **Reps** | **Mean LnP(K)** | **Stdev LnP(K)** | **Ln'(K)** | **\|Ln''(K)\|** | **Delta K** |
| --- | --- | --- | --- | --- | --- | --- |
| 1 | 6 | -31278.766667 | 0.051640 | — | — | — |
| 2 | 6 | -29852.550000 | 1.459795 | 1426.216667 | 734.116667 | **502.890416** |
| 3 | 6 | -29160.450000 | 181.506923 | 692.100000 | 39.216667 | 0.216062 |
| 4 | 6 | -28507.566667 | 4.380715 | 652.883333 | 333.033333 | 76.022592 |
| 5 | 6 | -28187.716667 | 10.365986 | 319.850000 | 37.550000 | 3.622424 |
| 6 | 6 | -27905.416667 | 27.452316 | 282.300000 | 103.983333 | 3.787780 |
| 7 | 6 | -27727.100000 | 9.888579 | 178.316667 | 17.100000 | 1.729268 |
| 8 | 6 | -27531.683333 | 19.181806 | 195.416667 | 143.433333 | 7.477572 |
| 9 | 6 | -27479.700000 | 242.410330 | 51.983333 | 190.016667 | 0.783864 |
| 10 | 6 | -27237.700000 | 20.848981 | 242.000000 | — | — |

S4 File Fig 8b. STRUCTURE output, Series 1 and Series 2, western Eurasia, England, Ireland and Wales, 6 SSRs, 1294 samples, 63 groups (25 England Ireland Wales Counties). NB Tipperary (Ire) is the only county with Series 2 >Series 1.
