# Supplementary figures and images for: DNA analysis of Castanea sativa (sweet chestnut) in Britain and Ireland: Elucidating European origins and genepool diversity
Source: PLoS One. 2019 Sep 25;14(9):e0222936. doi: 10.1371/journal.pone.0222936 (PMC6760806; doi:10.1371/journal.pone.0222936)

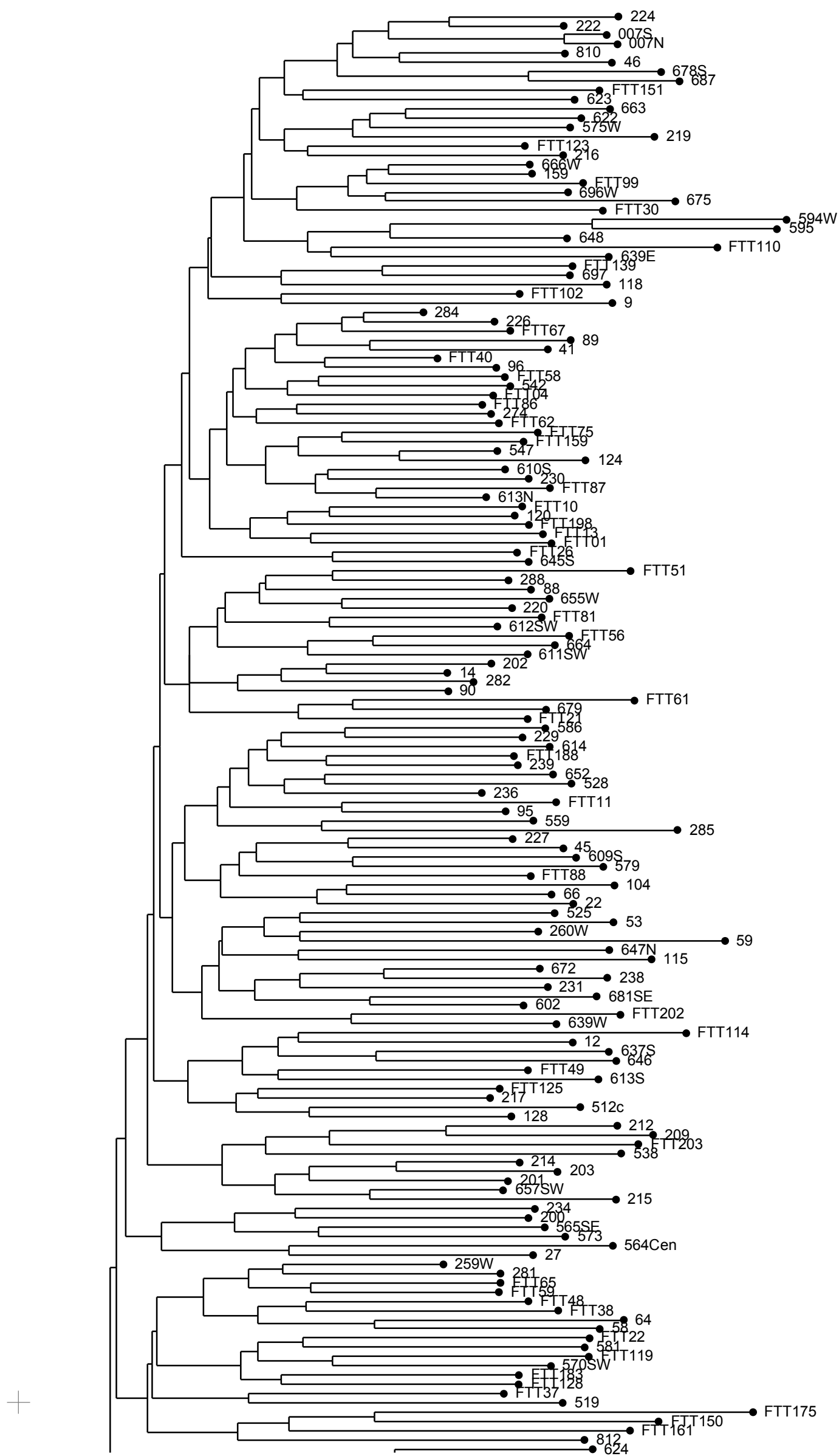

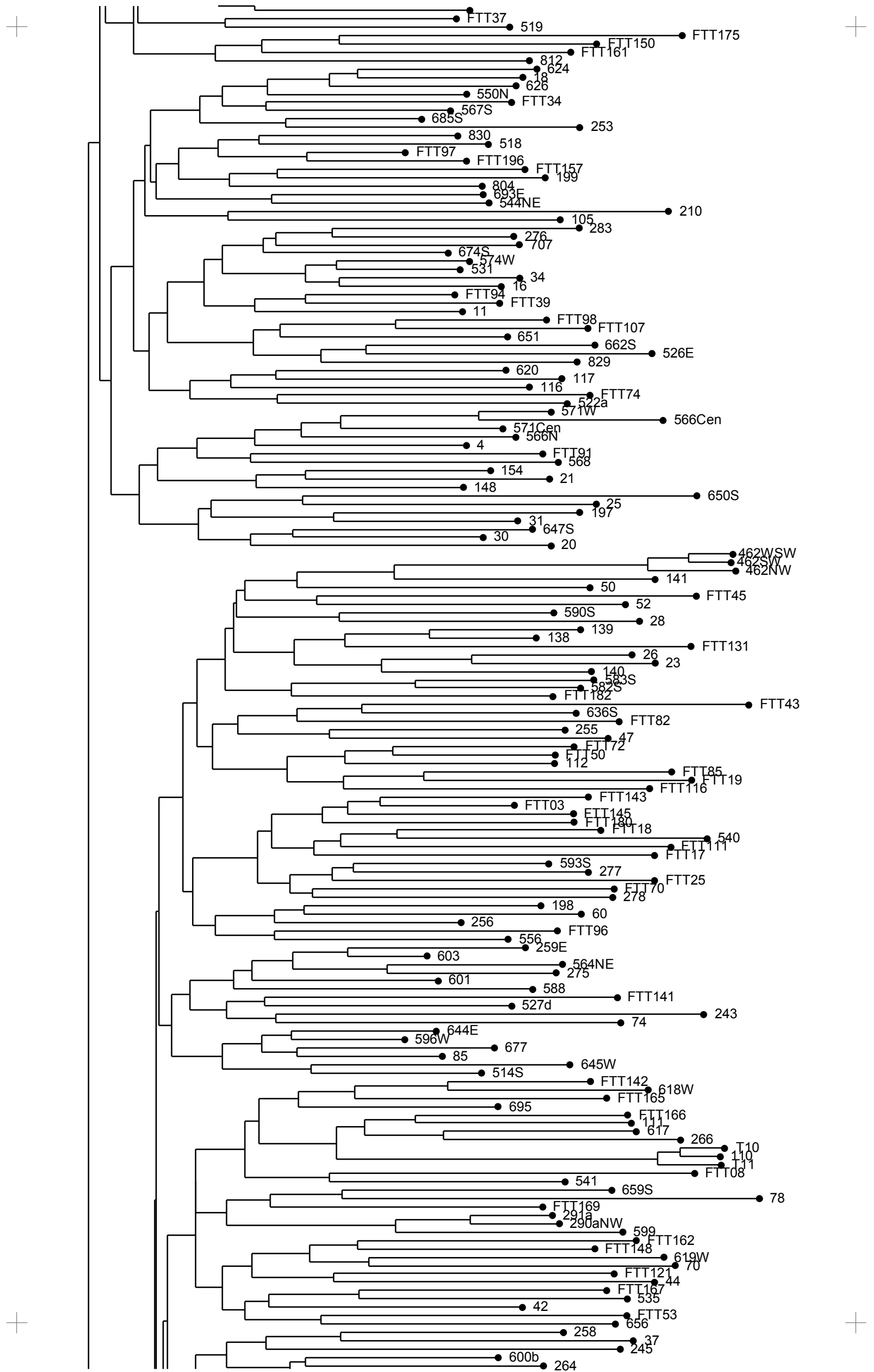

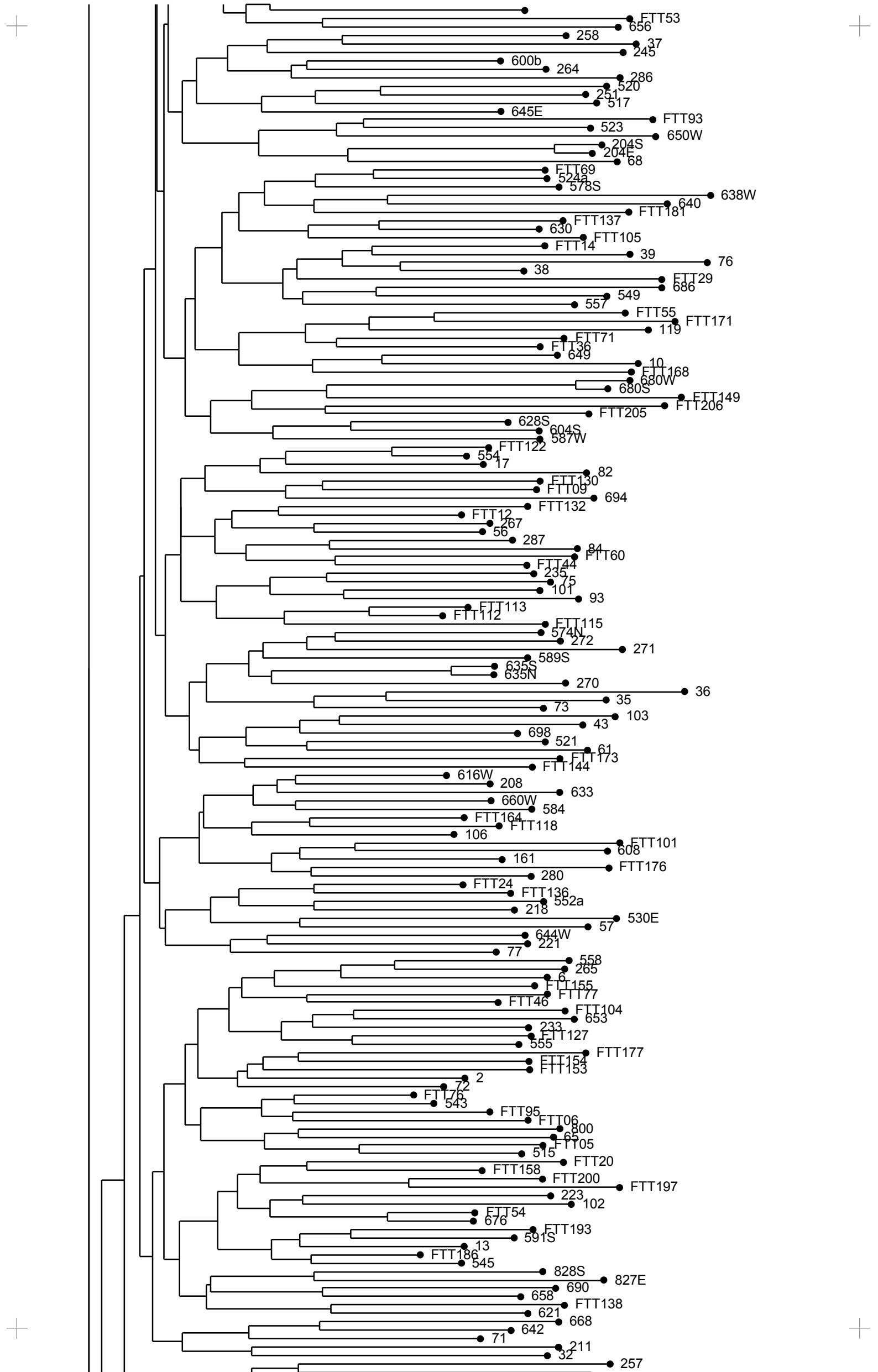

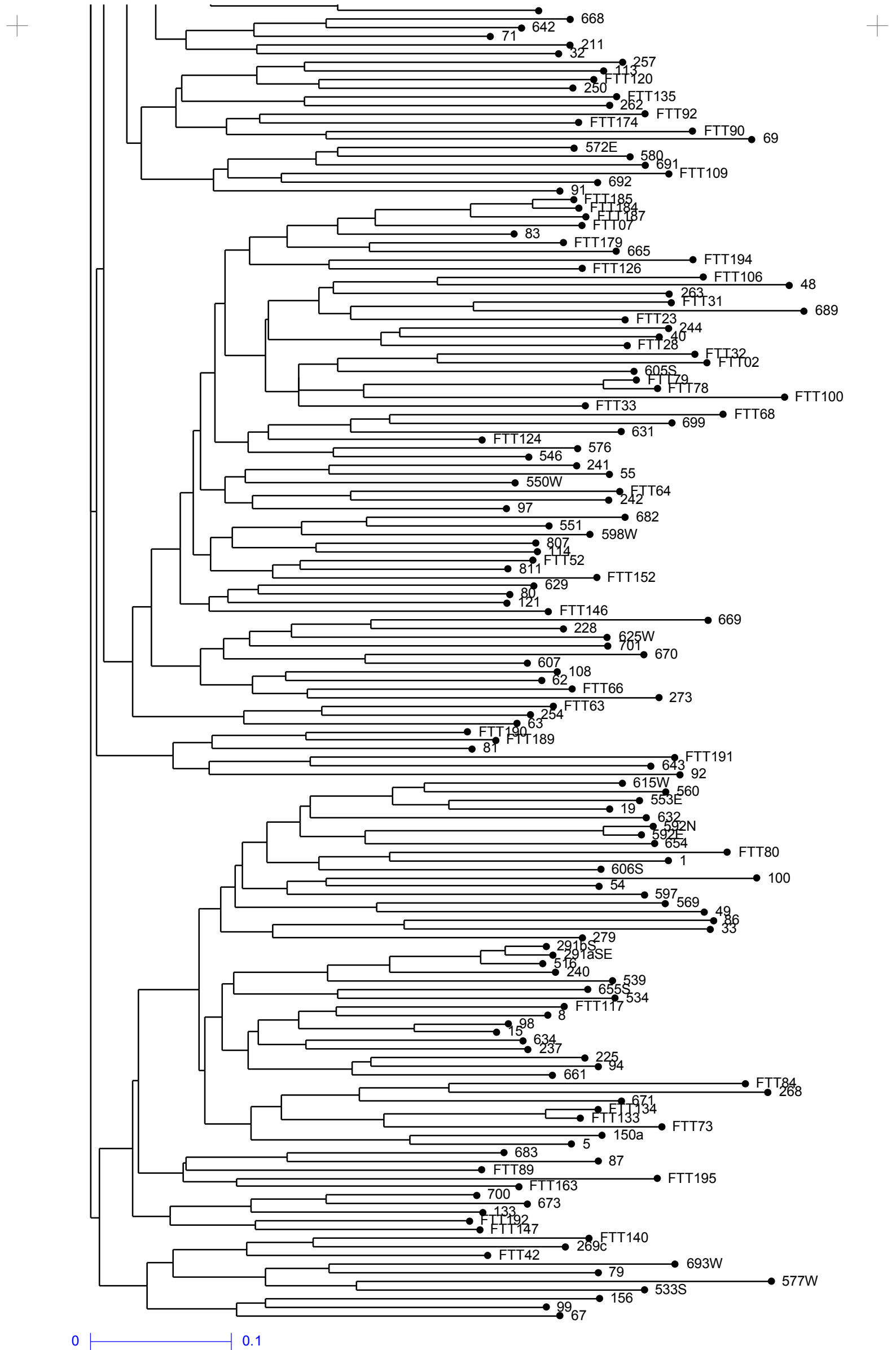

Supplement: S1 Fig — (PDF) [file pone.0222936.s006.pdf]
