# Supplementary material for: DNA analysis of Castanea sativa (sweet chestnut) in Britain and Ireland: Elucidating European origins and genepool diversity
Source: PLoS One. 2019 Sep 25;14(9):e0222936. doi: 10.1371/journal.pone.0222936 (PMC6760806; doi:10.1371/journal.pone.0222936)
Supplement: S2 Table — (DOCX) [file pone.0222936.s008.docx]

**S2 Table. AMOVA for England, Ireland and Wales**,

8 loci, 611 samples (GenAlEx 6.51)

| **Summary AMOVA Table** | | | | | |
| --- | --- | --- | --- | --- | --- |
| **Source** | **df** | **SS** | **MS** | **Est. Var.** | **%** |
| Among Pops | 2 | 13.508 | 6.754 | 0.022 | 1% |
| Among Indivs | 608 | 1869.427 | 3.075 | 0.129 | 4% |
| Within Indivs | 611 | 1721.000 | 2.817 | 2.817 | 95% |
| Total | 1221 | 3603.935 |  | 2.968 | 100% |
| **F-Statistics** | **Value** | **P** |  | | |
| F_ST_ | 0.007 | 0.001 |  |  |  |
| F_IS_ | 0.044 | 0.001 |  |  |  |
| F_IT_ | 0.051 | 0.001 |  |  |  |
