# Supplementary material for: DNA analysis of Castanea sativa (sweet chestnut) in Britain and Ireland: Elucidating European origins and genepool diversity
Source: PLoS One. 2019 Sep 25;14(9):e0222936. doi: 10.1371/journal.pone.0222936 (PMC6760806; doi:10.1371/journal.pone.0222936)
Supplement: S7 Table — (DOCX) [file pone.0222936.s013.docx]

**S7 Table. Genetic diversity values** of the 38 continental European populations with England, Ireland and Wales samples (6 SSRs, 1332 samples, 41 groups).

| **Group** | **N** | **Na** | **Ne** | **I** | **Ar** | **PAr** | **Ho** | **He** | **uHe** | **F_IS_**  **P<0.01** |
| --- | --- | --- | --- | --- | --- | --- | --- | --- | --- | --- |
| ENGLAND | 513 | 15.333 | 5.228 | 1.826 | 6.53 | 0.06 | 0.703 | 0.742 | 0.742 | 0.058 |
| IRELAND | 48 | 10.667 | 4.885 | 1.748 | 6.59 | 0.04 | 0.677 | 0.716 | 0.724 | 0.063 |
| WALES | 47 | 9.500 | 4.432 | 1.644 | 5.91 | 0.09 | 0.638 | 0.718 | 0.726 | 0.125 |
| PT01 | 17 | 5.500 | 3.660 | 1.351 | 4.64 | 0.07 | 0.676 | 0.686 | 0.707 | 0.010 |
| PT02 | 20 | 6.667 | 3.292 | 1.353 | 5.05 | 0 | 0.700 | 0.650 | 0.666 | -0.072 |
| PT03 | 20 | 6.167 | 3.725 | 1.426 | 5.07 | 0.11 | 0.758 | 0.686 | 0.704 | -0.083 |
| SP01 | 14 | 4.667 | 3.189 | 1.246 | 4.18 | 0.11 | 0.643 | 0.664 | 0.688 | 0.057 |
| SP02 | 15 | 4.833 | 3.629 | 1.342 | 4.5 | 0 | 0.800 | 0.694 | 0.718 | -0.160 |
| SP06 | 20 | 5.500 | 3.296 | 1.307 | 4.55 | 0 | 0.575 | 0.658 | 0.675 | 0.159 |
| SP04 | 26 | 5.833 | 3.162 | 1.299 | 4.47 | 0.06 | 0.526 | 0.654 | 0.667 | 0.196 |
| SP05 | 14 | 4.167 | 3.055 | 1.196 | 3.91 | 0.11 | 0.595 | 0.656 | 0.680 | 0.110 |
| SP06 | 14 | 5.500 | 3.368 | 1.321 | 4.83 | 0 | 0.595 | 0.654 | 0.678 | 0.092 |
| SP07 | 15 | 5.833 | 3.392 | 1.357 | 4.97 | 0.1 | 0.711 | 0.670 | 0.693 | -0.053 |
| SP08 | 29 | 5.167 | 3.122 | 1.263 | 4.26 | 0 | 0.603 | 0.652 | 0.663 | 0.059 |
| SP09 | 13 | 4.167 | 2.990 | 1.170 | 3.97 | 0 | 0.474 | 0.634 | 0.659 | 0.261 |
| SP10 | 15 | 4.167 | 3.043 | 1.186 | 3.89 | 0 | 0.756 | 0.645 | 0.667 | -0.186 |
| SP11 | 12 | 4.833 | 3.350 | 1.321 | 4.57 | 0 | 0.681 | 0.689 | 0.719 | 0.035 |
| SP12 | 11 | 5.167 | 3.809 | 1.327 | 4.99 | 0 | 0.606 | 0.641 | 0.672 | 0.050 |
| SP13 | 23 | 7.000 | 3.410 | 1.338 | 4.91 | 0.2 | 0.674 | 0.628 | 0.642 | -0.064 |
| SP14 | 14 | 5.667 | 4.074 | 1.500 | 5.31 | 0.01 | 0.690 | 0.732 | 0.759 | 0.053 |
| SP15 | 15 | 5.500 | 3.068 | 1.299 | 4.75 | 0.1 | 0.667 | 0.652 | 0.675 | -0.021 |
| SP16 | 15 | 5.333 | 3.462 | 1.285 | 4.63 | 0.16 | 0.600 | 0.641 | 0.663 | 0.068 |
| SP17 | 35 | 6.500 | 3.212 | 1.309 | 4.64 | 0.04 | 0.557 | 0.628 | 0.637 | 0.085 |
| FR01 | 15 | 5.500 | 3.808 | 1.266 | 4.82 | 0.09 | 0.667 | 0.597 | 0.617 | -0.124 |
| FR02 | 16 | 6.667 | 3.820 | 1.400 | 5.54 | 0.03 | 0.521 | 0.634 | 0.654 | 0.229 |
| FR03 | 9 | 4.333 | 3.458 | 1.249 | 4.33 | 0 | 0.593 | 0.655 | 0.694 | 0.133 |
| IT01 | 26 | 7.000 | 4.151 | 1.526 | 5.53 | 0.11 | 0.628 | 0.698 | 0.712 | 0.102 |
| IT02 | 25 | 7.000 | 3.436 | 1.414 | 5.08 | 0.06 | 0.560 | 0.673 | 0.687 | 0.196 |
| IT03 | 25 | 8.500 | 4.475 | 1.648 | 6 | 0.13 | 0.767 | 0.741 | 0.756 | -0.048 |
| IT04 | 26 | 5.667 | 2.984 | 1.121 | 4.01 | 0.04 | 0.526 | 0.557 | 0.568 | 0.063 |
| IT05 | 23 | 8.333 | 3.909 | 1.535 | 5.82 | 0.2 | 0.630 | 0.684 | 0.699 | 0.100 |
| IT06 | 19 | 7.333 | 4.935 | 1.645 | 6 | 0.01 | 0.693 | 0.743 | 0.763 | 0.124 |
| IT07 | 26 | 6.000 | 2.835 | 1.201 | 4.27 | 0.12 | 0.577 | 0.603 | 0.615 | 0.086 |
| IT08 | 26 | 7.500 | 4.610 | 1.643 | 5.65 | 0.01 | 0.731 | 0.766 | 0.781 | 0.059 |
| IT09 | 26 | 6.500 | 3.834 | 1.471 | 5.04 | 0 | 0.679 | 0.712 | 0.726 | 0.058 |
| SK02 | 22 | 4.167 | 2.257 | 0.947 | 3.48 | 0.07 | 0.568 | 0.512 | 0.524 | -0.091 |
| SK03 | 19 | 5.500 | 3.108 | 1.274 | 4.4 | 0.3 | 0.553 | 0.654 | 0.671 | 0.146 |
| HU01 | 30 | 5.667 | 3.301 | 1.350 | 4.55 | 0 | 0.633 | 0.679 | 0.691 | 0.060 |
| RO01 | 11 | 6.333 | 4.341 | 1.514 | 5.91 | 0.36 | 0.682 | 0.712 | 0.746 | 0.067 |
| RO02 | 11 | 4.000 | 2.760 | 1.011 | 3.81 | 0.21 | 0.424 | 0.532 | 0.557 | 0.176 |
| RO03 | 12 | 3.833 | 2.825 | 1.092 | 3.72 | 0.13 | 0.569 | 0.597 | 0.623 | 0.077 |

N = Number of samples, Na = Number of different alleles, Ne = Number of effective alleles, I = Shannon’s diversity index, Ar = Allelic richness, PAr = Private allelic richness, Ho = Observed heterozygosity, He = expected heterozygosity, uHe = Unbiased expected heterozygosity, F_IS_  = inbreeding coefficient. Ar and PAr calculated using 18 genes.
