# Supplementary material for: DNA analysis of Castanea sativa (sweet chestnut) in Britain and Ireland: Elucidating European origins and genepool diversity
Source: PLoS One. 2019 Sep 25;14(9):e0222936. doi: 10.1371/journal.pone.0222936 (PMC6760806; doi:10.1371/journal.pone.0222936)
Supplement: S10 Table — (DOCX) [file pone.0222936.s016.docx]

**S10 Table**. **F_ST_ threshold values** for differentiation of ‘high’ and ‘low’ in continental European with England, Ireland Wales datasets, estimated as 10 and 90 percentiles in each dataset. The fields in *italics* refer to the England, Ireland, Wales Site Characterisation datasets.

| **Dataset group** | **10%ile**  **F_ST_ value** | **90%ile**  **F_ST_ value** |
| --- | --- | --- |
| continental European sites and 3 England, Ireland, Wales (41) | 0.0684 | 0.2264 |
| continental European and 77 England, Ireland, Wales sites (115) | 0.0501 | 0.216 |
| continental European and 245 England, Ireland, Wales sites (283) | 0.0243 | 0.2278 |
| *continental European and England, Ireland, Wales Counties* | *0.0427* | *0.1715* |
| *continental European and England, Ireland, Wales Seed Zones* | *0.0417* | *0.1620* |
| *continental European and England, Ireland, Wales Site Types* | *0.0448* | *0.1524* |
